# Real-time quality control of optical backscattering data from Biogeochemical-Argo floats

**DOI:** 10.12688/openreseurope.15047.2

**Published:** 2023-05-30

**Authors:** Giorgio Dall'Olmo, Udaya Bhaskar TVS, Henry Bittig, Emmanuel Boss, Jodi Brewster, Hervé Claustre, Matt Donnelly, Tanya Maurer, David Nicholson, Violetta Paba, Josh Plant, Antoine Poteau, Raphaëlle Sauzède, Christina Schallenberg, Catherine Schmechtig, Claudia Schmid, Xiaogang Xing

**Affiliations:** 1 Sezione di Oceanografia, Istituto Nazionale di Oceanografia e di Geofisica Sperimentale - OGS, Borgo Grotta Gigante, Trieste, 34010, Italy; 2 Plymouth Marine Laboratory, Plymouth, PL1 3DH, UK; 3 Indian National Centre for Ocean Information Services (INCOIS), Ministry of Earth Sciences (MoES), Ocean Valley, Pragathinagar (BO), Nizampet (SO), Hyderabad, 500090, India; 4 Leibniz Institute for Baltic Sea Research Warnemünde, Rostock-Warnemünde, 18119, Germany; 5 School of Marine Sciences, University of Maine, Orono, ME, 04469-5706, USA; 6 Cooperative Institute for Marine and Atmospheric Studies, University of Miami, Miami, FL, USA; 7 Laboratoire d’Océanographie de Villefranche (LOV), CNRS & Sorbonne University, Villefranche-sur-Mer, 06230, France; 8 British Oceanographic Data Centre (BODC), National Oceanography Centre (NOC), Liverpool, UK; 9 Monterey Bay Aquarium Research Institute (MBARI), Moss Landing, CA, 95039, USA; 10 Woods Hole Oceanographic Institution, Woods Hole, MA, 02478, USA; 11 Institute for Marine and Antarctic Studies, University of Tasmania, Hobart, TAS, Australia; 12 OSU Ecce Terra, CNRS & Sorbonne University, Paris Cedex 05, 75252, France; 13 NOAA/AOML/PHOD, Miami, FL, 33149, USA; 14 State Key Laboratory of Satellite Ocean Environment Dynamics, Second Institute of Oceanography, Hangzhou, China

**Keywords:** BGC Argo, BBP, oprical backscattering, particles

## Abstract

Background: Biogeochemical-Argo floats are collecting an unprecedented number of profiles of optical backscattering measurements in the global ocean. Backscattering (BBP) data are crucial to understanding ocean particle dynamics and the biological carbon pump. Yet, so far, no procedures have been agreed upon to quality control BBP data in real time.

Methods: Here, we present a new suite of real-time quality-control tests and apply them to the current global BBP Argo dataset. The tests were developed by expert BBP users and Argo data managers and have been implemented on a snapshot of the entire Argo dataset.

Results: The new tests are able to automatically flag most of the “bad” BBP profiles from the raw dataset.

Conclusions: The proposed tests have been approved by the Biogeochemical-Argo Data Management Team and will be implemented by the Argo Data Assembly Centres to deliver real-time quality-controlled profiles of optical backscattering. Provided they reach a pressure of about 1000 dbar, these tests could also be applied to BBP profiles collected by other platforms.

## Introduction

The optical backscattering coefficient quantifies the fraction of incident power that is scattered in the backward direction per unit pathlength, when an infinitesimally small water sample is illuminated by a collimated and monochromatic beam of light ( Mobley, 2022). In practice, the total volume scattering function, *β*( *θ*, *λ*), i.e., the fraction of incident power that is scattered at a given angle, *θ*, is measured at a given wavelength in vacuo, *λ*, and then used to derive the volume scattering function of particles, *β _p_* ( *θ*, *λ*), by subtracting the contribution of pure seawater, *β _sw_* ( *θ*, *λ*, *T*, *S*, *P*), that also depends on temperature, *T*, salinity, *S*, and (weakly) on pressure, *P* ( Hu *et al.*, 2019; Zhang & Hu, 2009; Zhang *et al.*, 2009):


$$\beta_{p}(\theta ,\;\lambda )=\beta (\theta ,\;\lambda )-\beta_{sw}(\theta ,\lambda ,T,S,P).\;(1)$$

Finally, *β _p_* is converted into the particle backscattering coefficient as follows:


$$b_{bp}(\lambda )=2\pi \chi_{p}\beta_{p}(\theta ,\lambda ),\;(2)$$

where 2 *π* accounts for the azimuthal integration of the backscattered beam (assumed symmetrical), and *χ _p_* for the conversion between the volume scattering function by particles at a given angle and its integral in the backward direction ( Boss & Pegau, 2001; Oishi, 1990). While *b _bp_* ( *λ*) is the standard symbol used in marine optics to indicate particulate optical backscattering at a given wavelength, the BGC-Argo variable used to indicate this quantity is BBP. We will therefore use BBP in this manuscript that focuses on BGC-Argo data. BBP and *b _bp_* are however the same quantity.

BBP measurements and the quantities that can be derived from them are needed to improve our understanding of ocean ecosystems and biogeochemical cycles. BBP is correlated with the concentration of particulate organic carbon ( Cetinić *et al.*, 2012; Koestner *et al.*, 2022; Rasse *et al.*, 2017; Stramski *et al.*, 2008) and, near the surface, of phytoplankton carbon ( Graff *et al.*, 2015; Martinez-Vicente *et al.*, 2013) and particulate inorganic carbon ( Balch *et al.*, 1996; Terrats *et al.*, 2020). Spikes in BBP profiles have also been used to detect large, fast-sinking aggregates ( Briggs *et al.*, 2011; Briggs *et al.*, 2020) or animals that may be attracted to the light emitted by the sensor ( Haëntjens *et al.*, 2020). Finally, BGC-Argo data provide a means to validate remote-sensing BBP algorithms ( Bisson *et al.*, 2019).

So far more than 600 BGC-Argo floats have been equipped with optical backscattering sensors, and ~250 of them are currently active. Argo’s objective is to sustain 1000 operational six-variable BGC-Argo floats in the global ocean ( Claustre *et al.*, 2020; Roemmich *et al.*, 2019). With strong international collaboration and the recent launch of new BGC-Argo float programmes, such as the Global Ocean Biogeochemistry ( GO-BGC) array, the value of the global BGC-Argo BBP dataset will continue to expand.

The procedure to estimate BBP from different sensors with varying optical designs is standardised in the Argo data system - see here. As with other Argo parameters, BBP data are delivered via two data streams: “Real-Time” (RT) and “Delayed-Mode” (DM), see Argo Data Management Handbook.

Real-Time data should be delivered to users in less than 24 hours of the floats reaching the sea surface. In the Real-Time data stream, only automated quality-control checks can be applied to flag obviously bad data ( Bittig *et al.*, 2019). These checks are needed to allow non-experts (e.g., operational modellers) to exploit the Argo BBP data in real time. Delayed-Mode quality control is meant to provide the best-quality data for scientific applications. It is carried out in discrete time intervals of months to years, because it requires operators to implement tests that include comparisons with climatologies or analyses in a multiparameter context.

To deliver these two data streams, the Argo community has been developing common procedures for each of the variables measured. However, presently, the BGC-Argo programme has not officially released any document specific to the BBP parameter describing quality-control procedures (RT or DM). The general Argo Quality Control Manual for Biogeochemical Data version 1.0 lists two tests for BBP (Global-Range and Spike tests) that are now obsolete, given the new tests presented in this work.

The main motivation behind this work is therefore to deliver in real time a quality-controlled BBP dataset that can be used by non-experts interested in retrieving information on suspended particles from the BGC-Argo dataset. The objective of this manuscript is to present a new suite of BBP Real-Time Quality-Control (RTQC) tests, the methodology used to devise them, and the results of implementing them on the entire BGC-Argo BBP dataset. Delayed-Mode Quality-Control procedures are not developed herein, although this document may serve to pave the road for future BBP Delayed-Mode procedures. This work builds on a preliminary set of results from the Euro-Argo Rise project that were presented as a report.

## Data and methods

### Philosophy behind BBP RTQC tests

All BGC-Argo parameter data are paired with numeric flags that describe their quality (see Table 1 and reference table 3.2 in the Argo user’s manual). Given the audience that is expected to use the RTQC BBP dataset (i.e., non experts), the new tests presented in this document should be considered as “conservative”. In other words, these tests were tuned specifically to screen most profiles with questionable data, but may also occasionally flag data that are of good quality. To avoid flagging potentially good data as bad, the BBP-RTQC team agreed to use a quality-control flag equal to 3 (i.e., “probably bad” data), which should be interpreted as “do not use these data until an expert has checked them” ( Table 1). We therefore anticipate that the “Delayed-Mode Quality Control” of BBP should start by assessing the results of the RTQC tests for each float, following the example of what is done for the core-Argo mission - see here.

**Table 1.** Argo quality flags used in this work. Argo flags between 5 and 8 are not used in this work (see Argo User’s Manual).

QC flag	Meaning
1	“Good data”: All real-time QC tests passed
2	“Probably good data”: These data are to be used with caution
3	“Probably bad data”: Do not use until an expert has checked these data
4	“Bad data”: Do not use these data
9	“Missing data”

The Argo Data Assembly Centres (DACs) have the responsibility of implementing these tests and then submitting the quality-controlled data to the Argo Global Data Assembly Centres (GDACs). To minimise the impact of implementing these tests on the resource-limited DACs 1) tests were kept simple to ease implementation; 2) the number of tests was kept to a minimum; 3) all relevant code was made available; and 4) examples of input and expected output for each test were provided.

### Approach

To define the new BBP RTQC tests, we followed an iterative process. Tests were initially applied to a random subset of Argo “B-files” (i.e., containing the raw BBP profiles) extracted from the GDAC dataset (~60 floats from different DACs, covering different ocean regions and different model floats, snapshot from December 2021) and results were visually checked to refine the tests. Visual checks included (i) identifying anomalous profiles based on expert knowledge (e.g., expected range of BBP values at depth and at the surface, expected shape of the profile, negative BBP values) and (ii) verifying that the newly developed tests flagged anomalous values. These preliminary tests were then applied to the entire GDAC dataset ( 632 floats, snapshot from December 2021) and results assessed by the BGC-Argo community that contributed to the development of the quality control of BBP (i.e., the co-authors of this manuscript). Feedback included a request to minimise the efforts required by DACs to implement these tests and a suggestion to devise fewer and simpler tests. To further limit the overall number of tests to be implemented, an analysis of the overlap between tests was also requested. A revised suite of tests was developed and applied, and results again shared and discussed by means of a second on-line workshop. The tests were developed for BBP measured at a wavelength in vacuo of 700 nm (i.e., BBP700), but should be applicable to BBP measured at any other wavelength as well.

These interactions with the community allowed us to converge on a final suite of tests that was presented and agreed upon at the 22nd Argo Data Management Team meeting (Dec 2021) and should be implemented by the DACs. All code developed is written in an open programming language (Python) and shared through a dedicated Euro-Argo GitHub repository (the first author is responsible for this repository).

While the interactions with the community were crucial in defining the final test suite, they introduced a certain level of subjectivity in how the tests were selected. This subjectivity, rather than decreasing the value of the resulting tests, incorporates the knowledge of experts in optical backscattering and management of the Argo data stream. We therefore consider this decision step as fundamental in defining the final test suite.

All tests were applied independently of each other (no order was defined) and the statistics computed reflect this choice (i.e., the same data can be flagged by multiple tests). Tests were applied to all data at the GDAC even if profiles had been deemed bad by the DAC operators (i.e., “greylisted”, in Argo terminology).

To minimise overlap among tests, the fraction of data points flagged by all pairs of preliminary tests was calculated. Test overlapping was used to both screen the initial set of proposed tests discussed with the BGC-Argo community and to quantify the level of overlap between the final set of tests.

Due to the non-standard missions with which BGC-Argo floats were initially operated, most of the BGC-Argo BBP data collected so far (Argo snapshot of December 2021) have been measured in the upper 1000 dbar of the water column. Our tests therefore were largely based on data at pressures ≤1000 dbar. Nonetheless, when deeper data were available, the tests and resulting flags were applied to the full profile depth (29% of the analysed profiles had a maximum pressure ≥1900 dbar). Importantly, this assumes that the profile is collected in deep waters, and far from the bottom near which suspended sediments might invalidate the assumptions of some of the proposed tests (see also discussion on High-Deep-Value test). Pressure values were extracted from the variable “PRES” in the Argo B-files.

To smooth BBP profiles, a median filter with a window size of 11 points was used in some of the proposed tests.

## Results

In the following section, we present five new RTQC tests for BBP. The proposed tests were applied to a total of 68,815 profiles from 632 floats, representing all major ocean basins as well as the Mediterranean and Black Seas. The tests, presented in order of decreasing percentage of data points flagged, are: Missing-Data test, High-Deep-Value test, Noisy-Profile test, Negative-BBP test, and Parking-Hook test. This order could be used to define the sequence in which the tests are applied during RTQC. To help the reader interested in directly comparing this manuscript to the corresponding code, in the following text we present the names of specific parameters used in the code using different fonts (e.g., MIN_N_PERBIN).

Each test is presented with a common structure composed of six parts:

1. “ *Objective*”, presenting the purpose of the test;
2. “ *Example*”, a plot of one or more examples of problematic profiles targeted by the test;
3. “ *Implementation*”, explaining how to implement the test (see also related code at the GitHub repository);
4. “ *Flagging*”, describing what flags are used and how; and
5. “ *Flow chart*”, a figure describing the different steps required to implement the test;
6. “ *Results*”, summarising the results of implementing this test.

### Proposed BBP RTQC tests

***Missing-Data test*.** *Objective*: To detect and flag profiles that have a large fraction of missing data. Missing data could indicate shallow profiles (caused by a specific float mission and/or bathymetry) or incomplete profiles due to a malfunctioning sensor.

**Figure 1.**
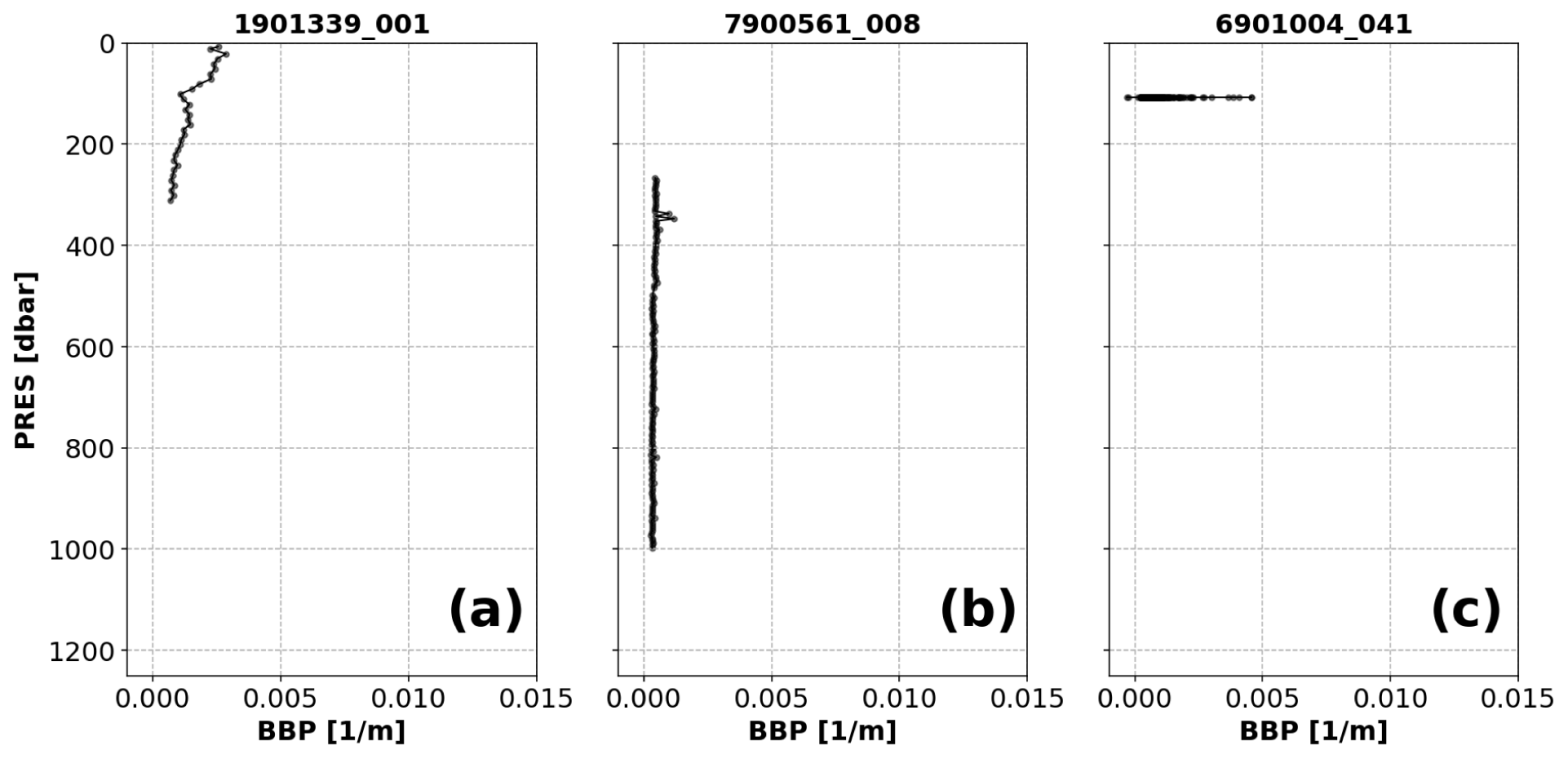
Examples of profiles flagged by the Missing-Data test. The titles of each subplot include the World Meteorological Organisation number of the Argo float and the number of the profile shown.

*Example*: See Figure 1.

*Implementation*: The upper 1000 dbar of the profile are divided into 10 pressure bins with the following lower boundaries (all in dbar): 50, 156, 261, 367, 472, 578, 683, 789, 894, 1000. For example, the first bin covers the pressure range [0, 50), the second [50, 156), etc. The test fails if any of the bins does not contain data points ( MIN_N_PERBIN = 1).

**Figure 2.**
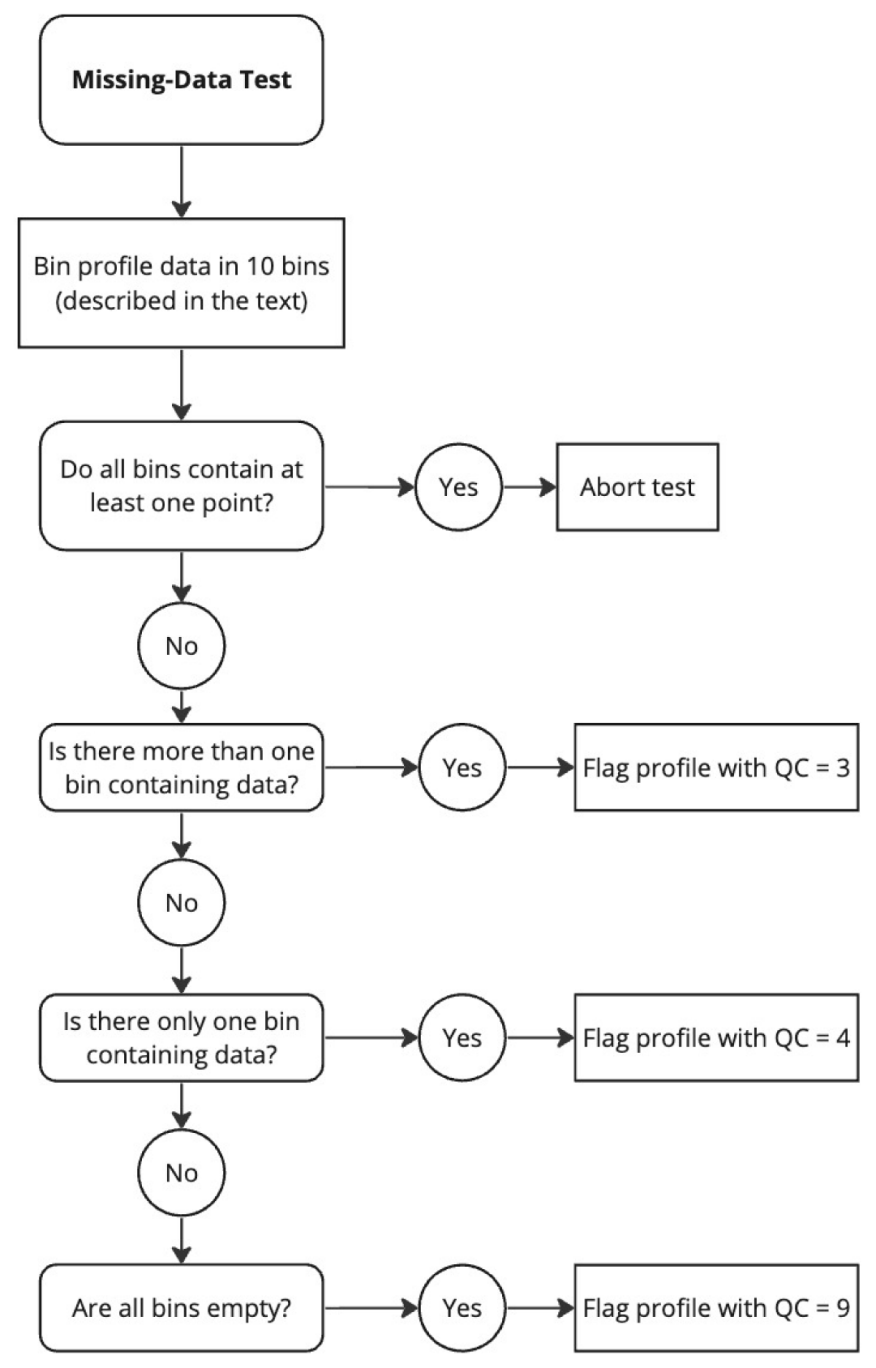
Flow chart for the Missing-Data test.

*Flagging*: Different flags are assigned depending on how many bins are empty. See flow chart in Figure 2.

(i) If there are bins with missing data, but the number of bins containing data is greater than one ( Figure 1a,b), then a QC flag of 3 is assigned to all BBP data in the profile (and the profile can be reviewed further in delayed-mode).
(ii) If only one bin contains data ( Figure 1c), a QC flag of 4 is applied to the entire profile. This condition may indicate a malfunctioning sensor or a problem with how the pressure values were assigned to BBP.
(iii) If the profile has no data at all, a QC flag of 9 is applied to the entire profile. This condition may indicate a malfunctioning sensor.

**Figure 3.**
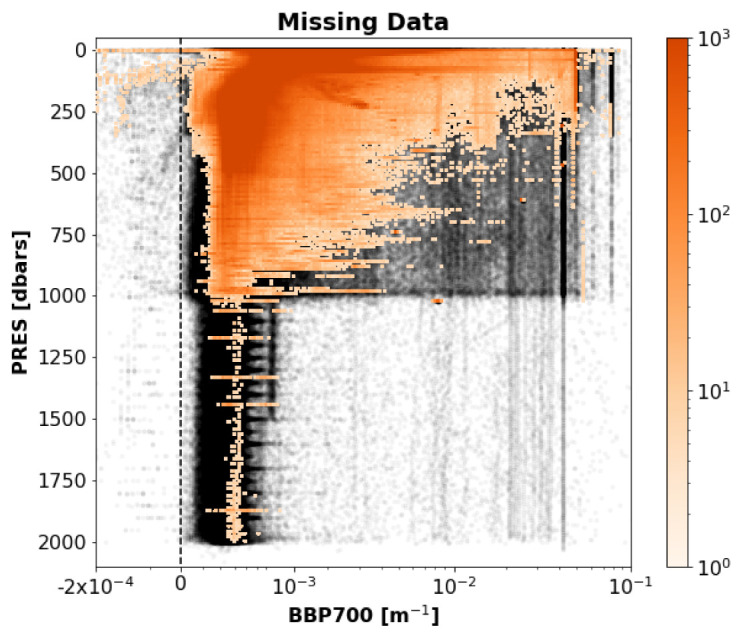
Two-dimensional histogram of the GDAC BBP data flagged by the Missing-Data test (colours represent the number of points in each bin; for clarity, only bins with at least 5 points are visualised). Black/grey points represent the rest of the analysed GDAC BBP data.

*Results*: This test flagged 10.7% of the analysed data in the GDAC ( Figure 3).

***High-Deep-Value test*.** *Objective*: To flag profiles with anomalously high BBP values at depth. High values at deeper depths could indicate a variety of problems, including biofouling, incorrect calibration coefficients, sensor malfunctioning. Note that high deep BBP values could also be valid data, for example in the case of sediment-resuspension events. A threshold value of 5 × 10 ^−4^ m ^−1^ was selected that is half of the value typical for surface BBP in the oligotrophic ocean ( Dall’Olmo *et al.*, 2012, e.g.,): median-filtered BBP data at depth are expected to be lower than this threshold value (typically ~ 2.5×10 ^−4^ m ^−1^) and with a peak-to-peak seasonal variability of < 1 × 10 ^−4^ m ^−1^; ( Poteau *et al.*, 2017).

**Figure 4.**
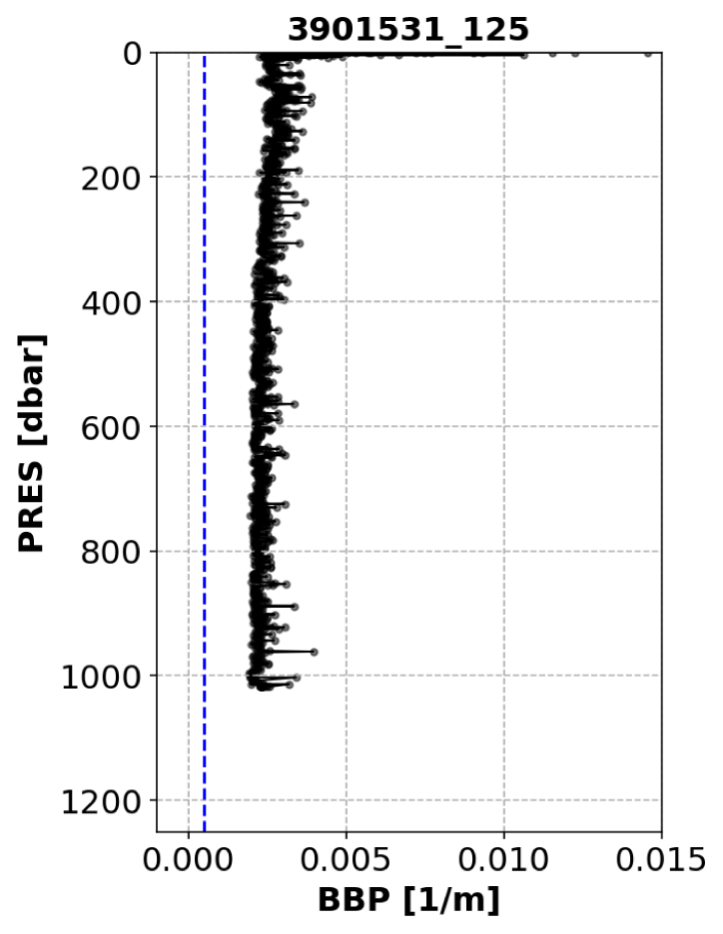
Example of profile flagged by the High-Deep-Value test. The blue dashed line represents the threshold above which the test fails. The title of the subplot includes the World Meteorological Organisation number of the Argo float and the number of the profile shown.

*Example*: See Figure 4.

*Implementation*: This tests fails if there is at least a certain number ( C_N_DEEP_POINTS = 5) of points deeper than a threshold depth ( C_DEPTH_THRESH = 700 dbar) and if the median of the median-filtered profile below C_DEPTH_THRESH is greater than a predefined threshold (i.e., C_DEEP_BBP700_THRESH = 0.0005 m ^−1^).

**Figure 5.**
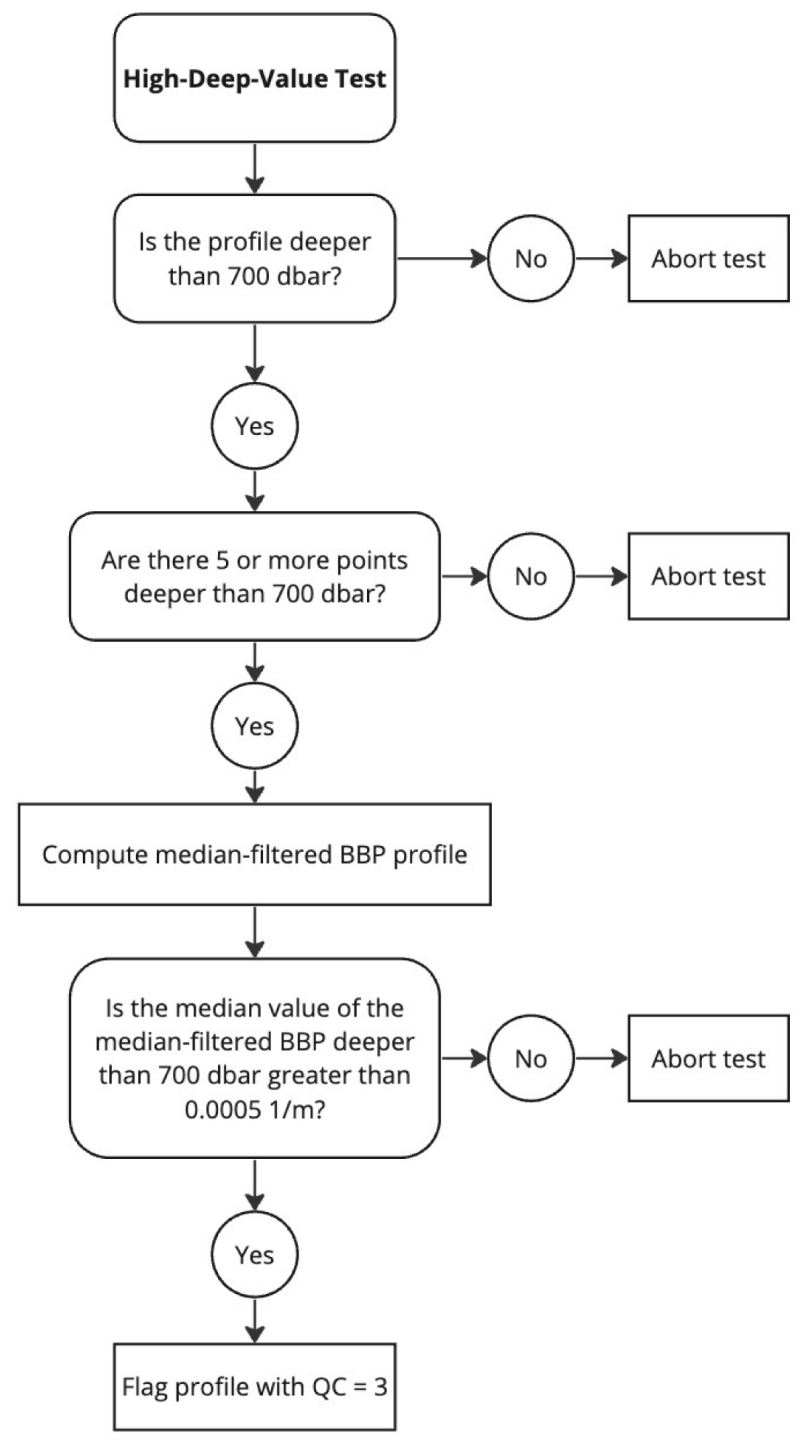
Flow chart for the High-Deep-Value test.

*Flagging*: If the test fails, a QC flag of 3 is applied to the entire profile. High deep BBP values can result from a variety of reasons, including natural causes. In the latter case, the quality flag could be set to“good data” during DMQC. See flow chart in Figure 5.

**Figure 6.**
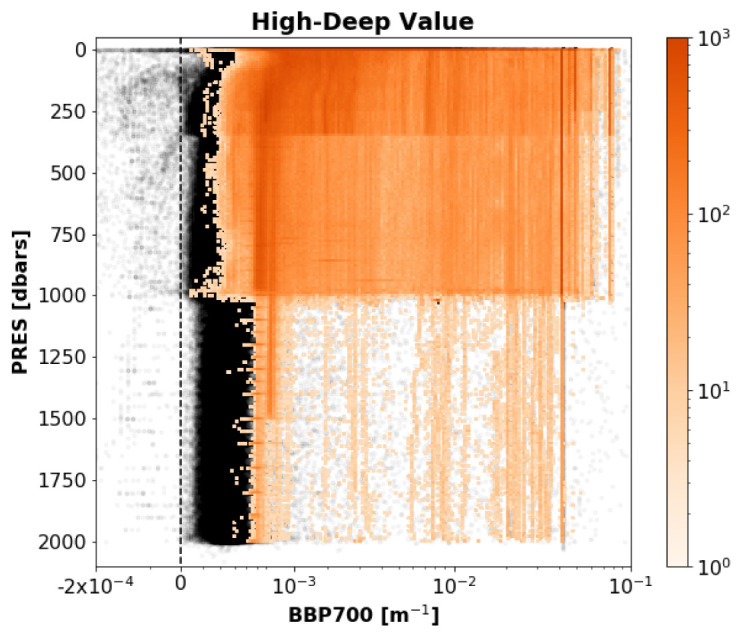
As Figure 3 but for the High-Deep-Value test.

*Results*: This test flagged 6.2% of the current data in the GDAC ( Figure 6).

***Noisy-Profile test*.** *Objective*: To flag profiles that are affected by noisy data. This noise could indicate sensor malfunctioning, clusters of BBP spikes caused by organisms attracted to the light emitted by the sensor ( Haëntjens *et al.*, 2020), or other anomalous conditions.

**Figure 7.**
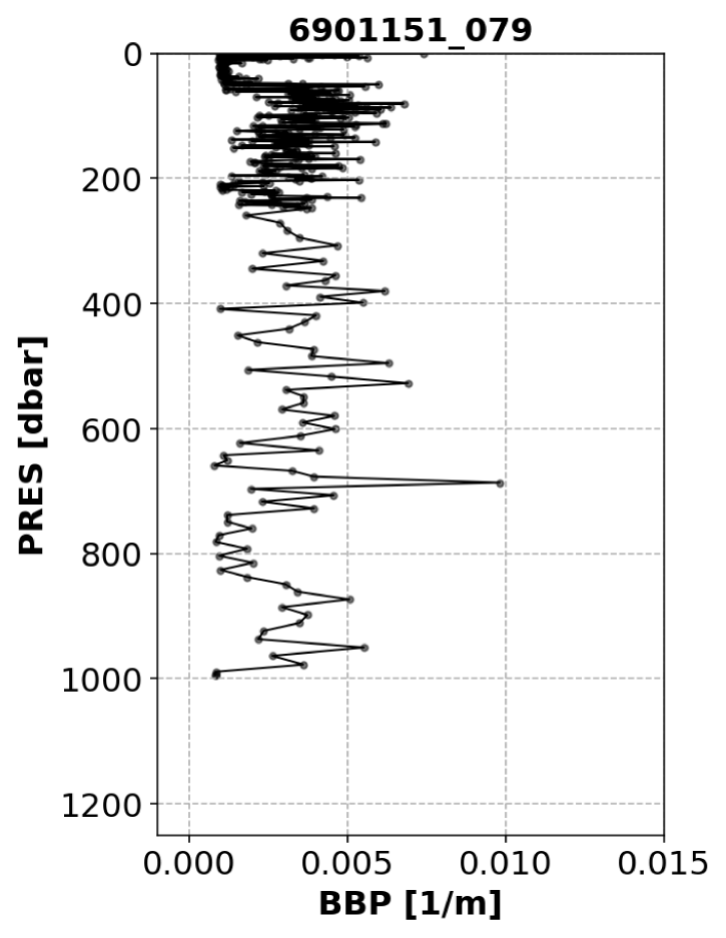
Example of a profile flagged by the Noisy-Profile test. The title of the subplot includes the World Meteorological Organisation number of the Argo float and the number of the profile shown.

*Example*: See Figure 7.

*Implementation*: The absolute residuals between the median-filtered BBP and the raw BBP values are computed below a pressure threshold B_PRES_THRESH = 100 dbar (this is to avoid surface data, where spikes are more common and generate false positives). Absolute residuals (instead of relative ones) were used to identify signals that are noisy compared to the expected values of BBP in the open ocean. The test fails if residuals with absolute values above a pre-defined threshold (i.e., B_RES_THRESHOLD = 0.0005 m ^−1^) occur in at least 10% of the profile data (i.e., B_FRACTION_OF_PROFILE_THAT_IS_OUTLIER = 0.10). These threshold values were selected after visual inspection of profiles from a subset of floats.

**Figure 8.**
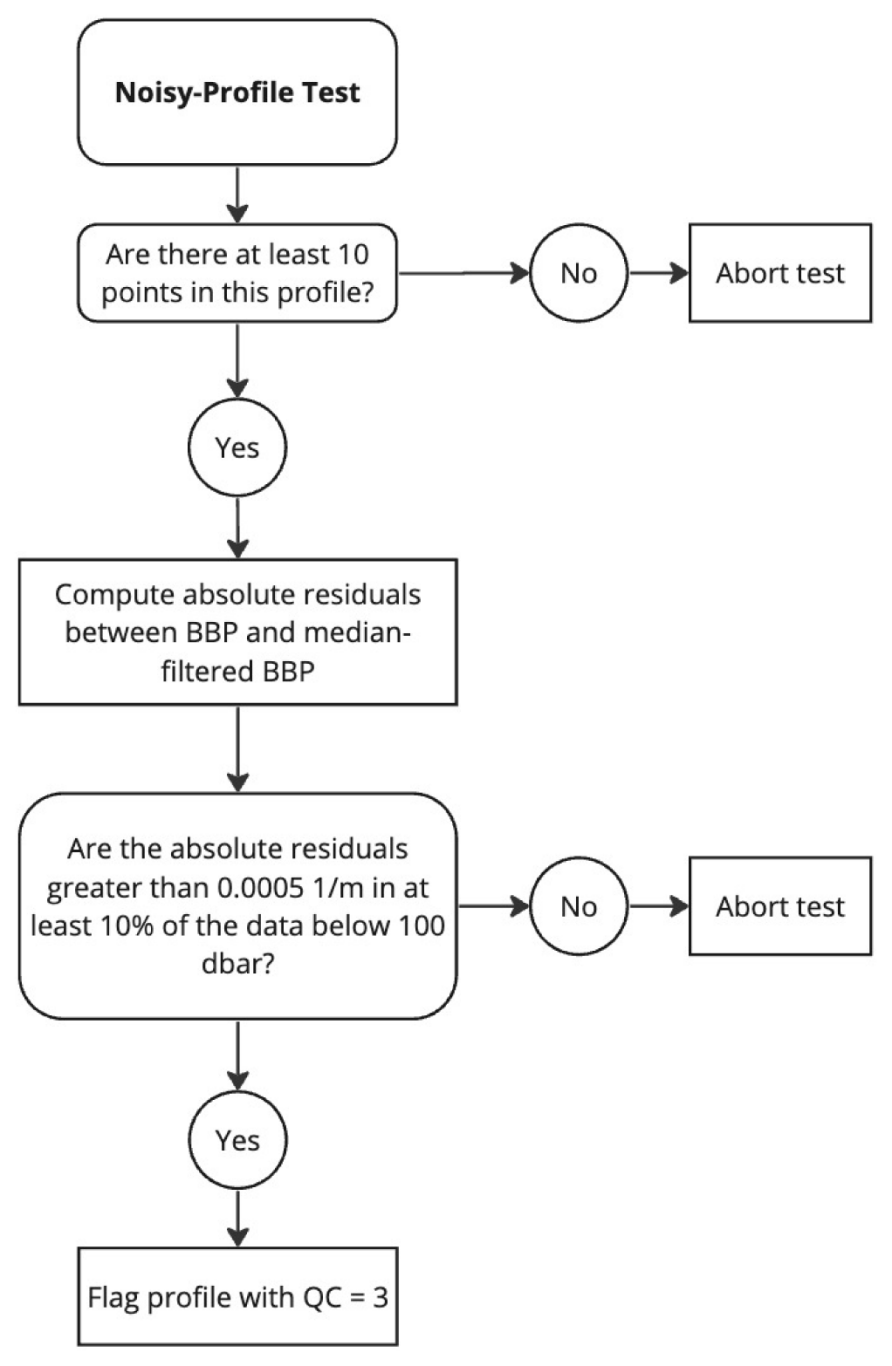
Flow chart for the Noisy-Profile test.

*Flagging*: If the test fails, a QC flag of 3 is assigned to the entire profile. See flow chart in Figure 8.

**Figure 9.**
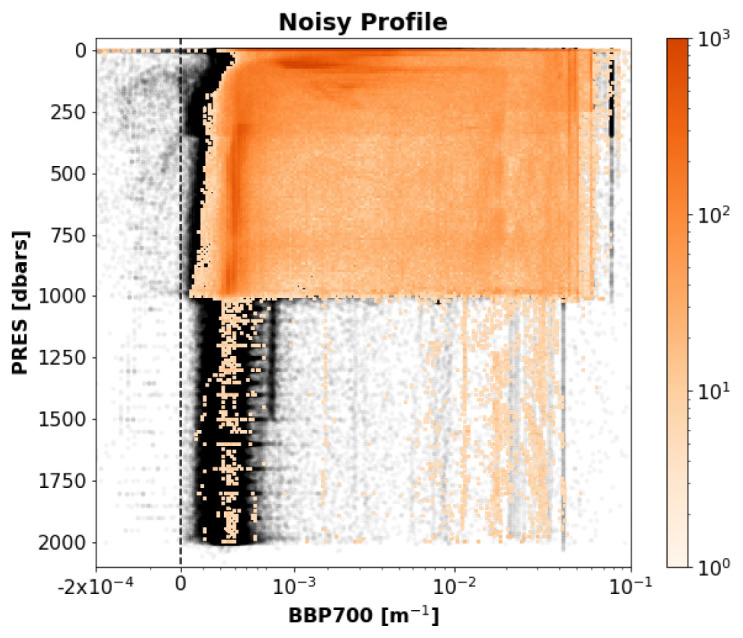
As Figure 3 but for the Noisy-Profile test.

*Results*: This test flagged 2.8% of the current data in the GDAC ( Figure 9).

***Negative-BBP test*.** *Objective*: To flag negative BBP values due to a variety of reasons including: sensor drift or malfunctioning, inaccurate calibration coefficients, or BBP sensor exposed to air.

**Figure 10.**
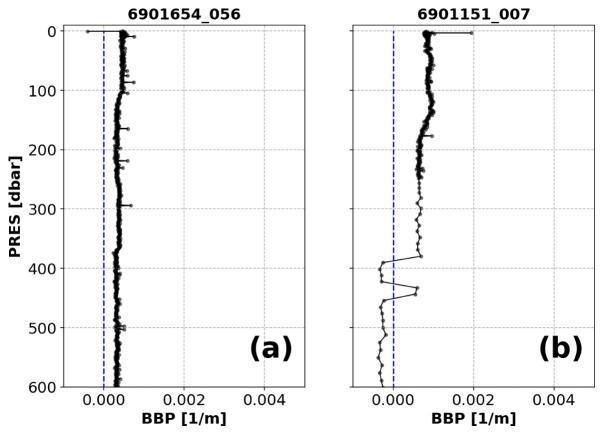
Examples of profiles flagged by the Negative-BBP test. ( **a**) Profile with negative BBP values only at pressures shallower than 5 dbar; ( **b**) profile with negative BBP values deeper than or at 5 dbar. The blue dashed lines represent the zero threshold beyond which the test fails. The title of the subplot includes the World Meteorological Organisation number of the Argo float and the number of the profile shown.

*Example*: See Figure 10.

*Implementation*: The test is implemented on the unfiltered BBP data.

**Figure 11.**
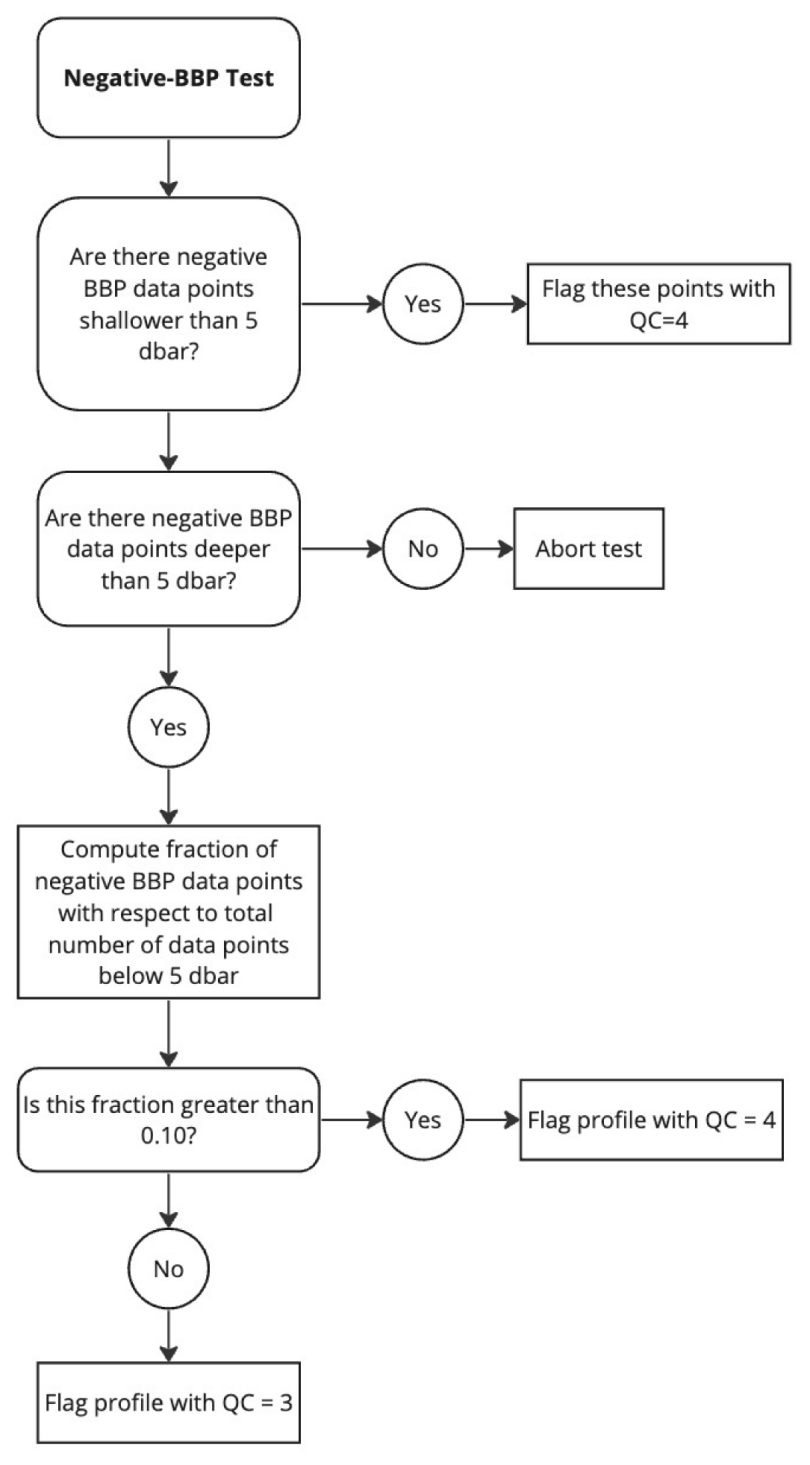
Flow chart for the Negative-BBP test.

*Flagging*: Different flagging is applied depending on whether the negative BBP values occur only near the surface (i.e., PRES < 5 dbar) or deeper in the water column (see flow chart in Figure 11):

(i) A QC flag of 4 is assigned to negative BBP points when these appear at pressures shallower than 5 dbar. This is used to flag negative BBP values near the surface that most likely represent data with a BBP sensor outside of the water.
(ii) To allow delayed-mode operators to requalify profiles with just a few deep negative points, at pressures greater than 5 dbar the flag is set depending on the fraction of negative BBP values with respect to the number of BBP measurements below 5 dbar. If the fraction of negative BBP values is greater than a pre-defined threshold (i.e., A_MAX_FRACTION_OF_BAD_POINTS = 0.10), then a QC flag of 4 is assigned to the entire profile.
(iii) Otherwise, a QC flag of 3 is assigned to the entire profile. BBP sensors that generate these deep negative BBP values are considered more at risk of malfunctioning and thus the entire profile is flagged.

**Figure 12.**
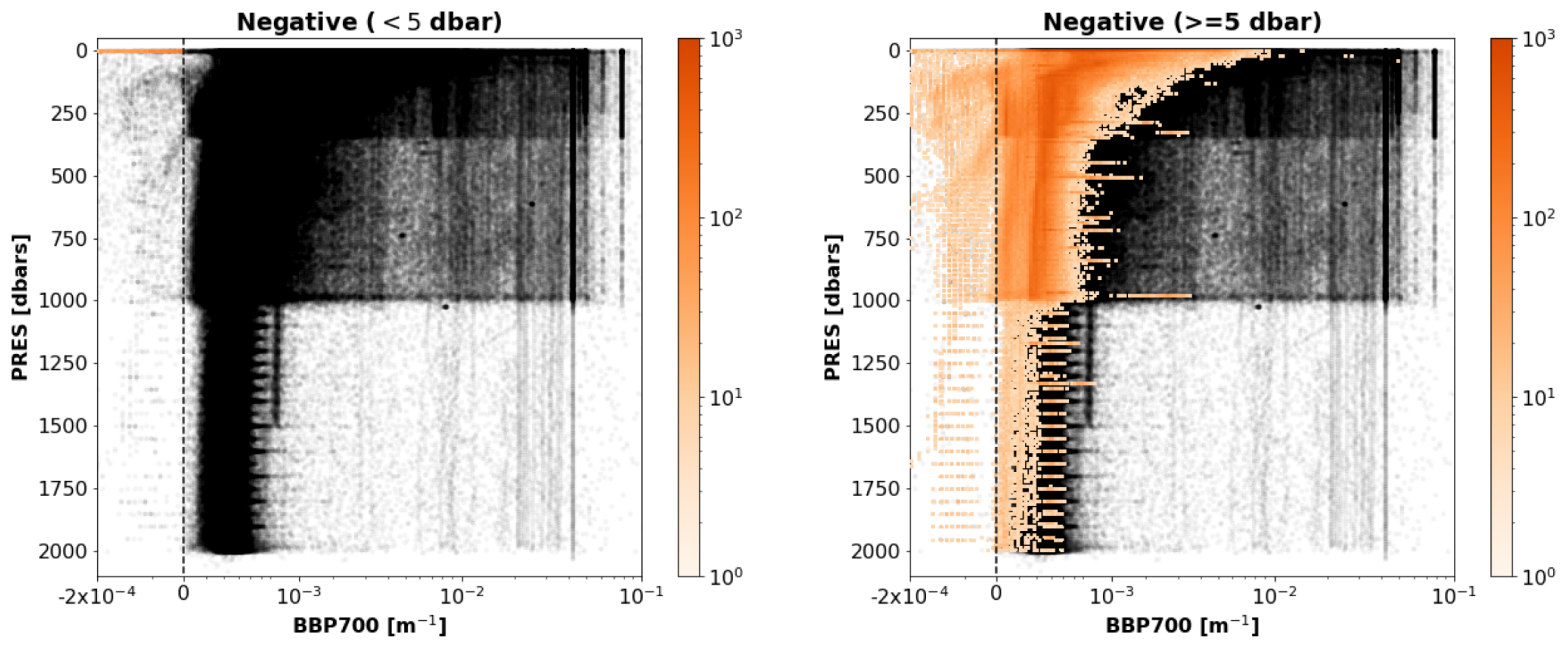
As Figure 3 but for the Negative-BBP test. Left plot: data with negative BBP values only at PRES < 5 dbar. Right plot: data with negative BBP values at PRES >= 5 dbar.

*Results*: This test flagged a total of 2.17% of the current data in the GDAC, 2.12% for negative BBP values deeper than or at 5 dbar and 0.05% for BBP values shallower than 5 dbar ( Figure 12).

***Parking-Hook test*.** *Objective*: When a float is drifting with the currents while at its parking pressure (typically 1000 dbar), particles may be depositing on the float and BBP sensor. These accumulated particles are likely released back into the water when the float descends to its maximum pressure (typically 2000 dbar), before starting the ascending profile during which data are collected. However, if the float does not descend to 2000 dbar before starting the BBP measurements, but immediately starts ascending towards the surface and measuring, then the accumulated particles might be measured by the BBP sensor as they are released back into the water. This is the likely cause of an increase in BBP at the start of the profile, when the parking pressure is close to the maximum pressure. The objective of this test is to flag these anomalous BBP points.

**Figure 13.**
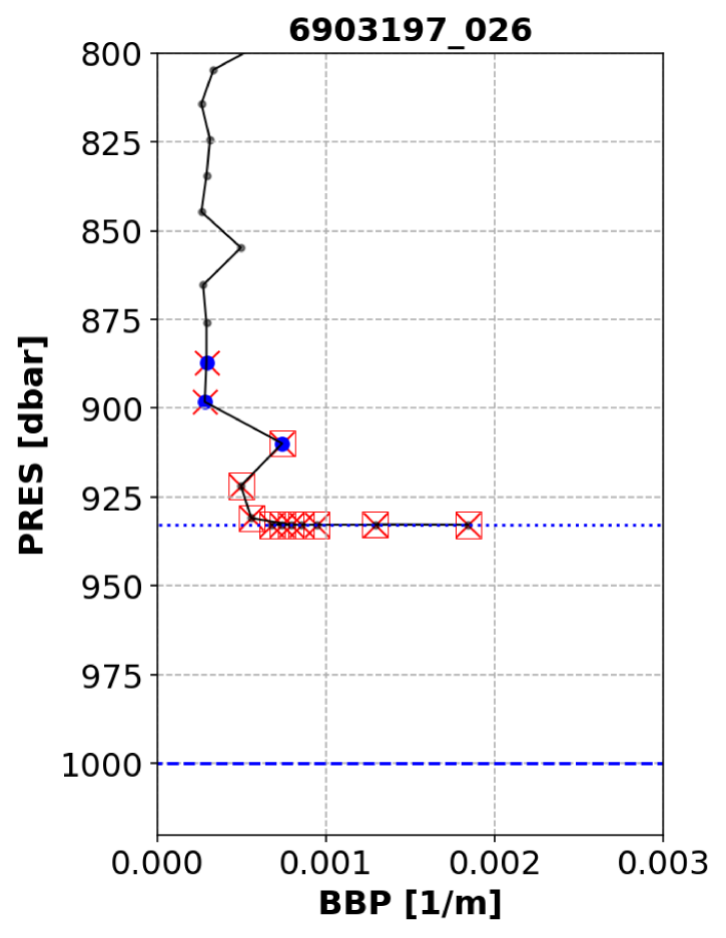
Example of profile flagged by the Parking-Hook test. The dashed and dotted blue lines represent the nominal parking pressure and actual maximum pressure recorded for this profile, respectively. Blue circles represent the points used to compute the baseline. Red crosses are the points to which the test is applied. Red squares are the points that failed the test. The title of the subplot includes the World Meteorological Organisation number of the Argo float and the number of the profile shown.

*Example*: See Figure 13.

*Implementation*: For ascending profiles, we first verify that the nearest BBP measurement above the maximum pressure recorded by the float ( maxPRES) is lower than a pre-defined threshold ( G_DELTAPRES2 = 20 dbar): if it is not, the test cannot be applied to this profile. This is to ensure that the baseline (computed below) is representative of the values of BBP at maxPRES. If the BBP measurement above maxPRES is less than 20 dbar away from it, we check that the profile starts from the parking pressure ( parkPRES, extracted from the mission configuration valid for the float cycle under exam) by testing that the absolute difference between the maxPRES and parkPRES is smaller than 100 dbar. If the profile does not start from the parking pressure, the test is aborted. If the profile starts from the parking pressure, a first pressure range is defined ( maxPRES - G_DELTAPRES2 > PRES >= maxPRES - G_DELTAPRES1, with G_DELTAPRES1 = 50 dbar, blue circles in Figure 13) over which a baseline is calculated as the median value of BBP augmented by a threshold value of 0.0002 m−1 (i.e., median(BBP) + G_DEV, with G_DEV = 0.0002 m−1). The test is then implemented over a second pressure range (i.e., PRES >= maxPRES - G_DELTAPRES1). The test fails if BBP within the second pressure range is greater than the baseline.

**Figure 14.**
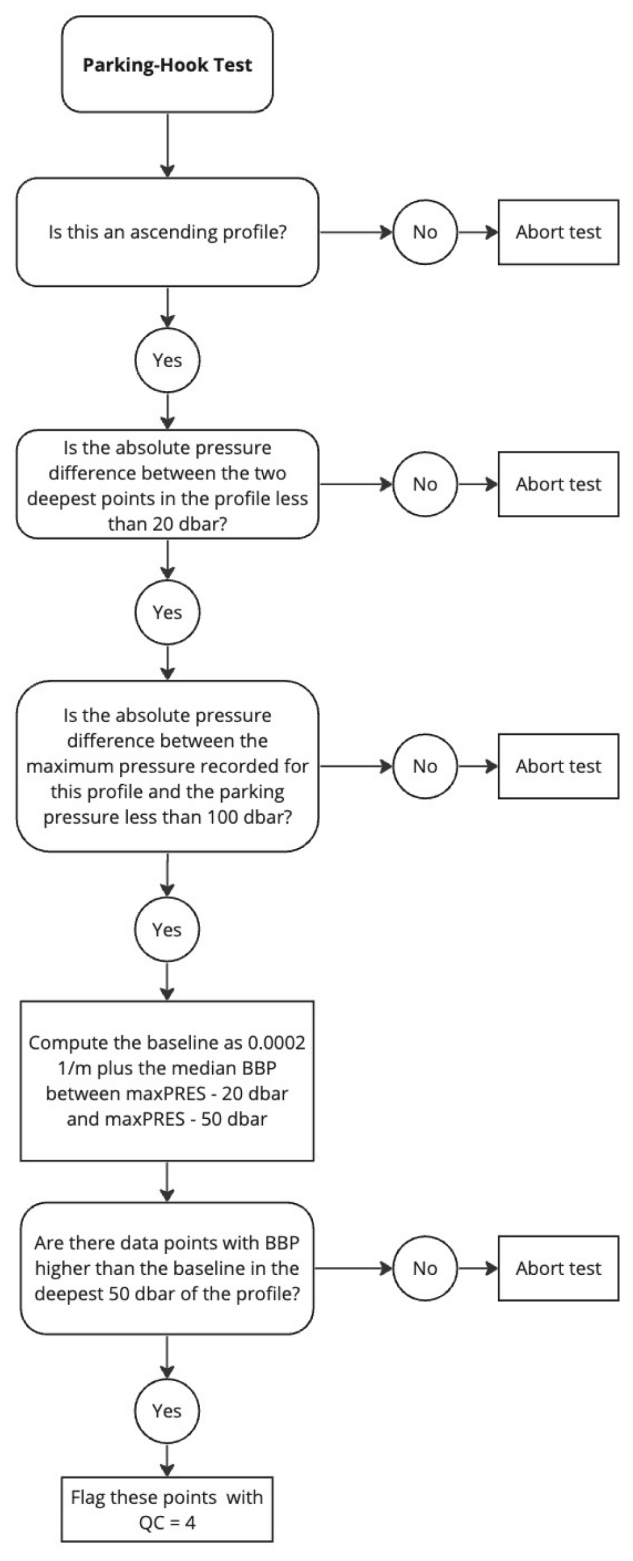
Flow chart for the Parking-Hook test.

*Flagging*: A QC flag of 4 is applied to the points that fail the test. See flow chart in Figure 14.

**Figure 15.**
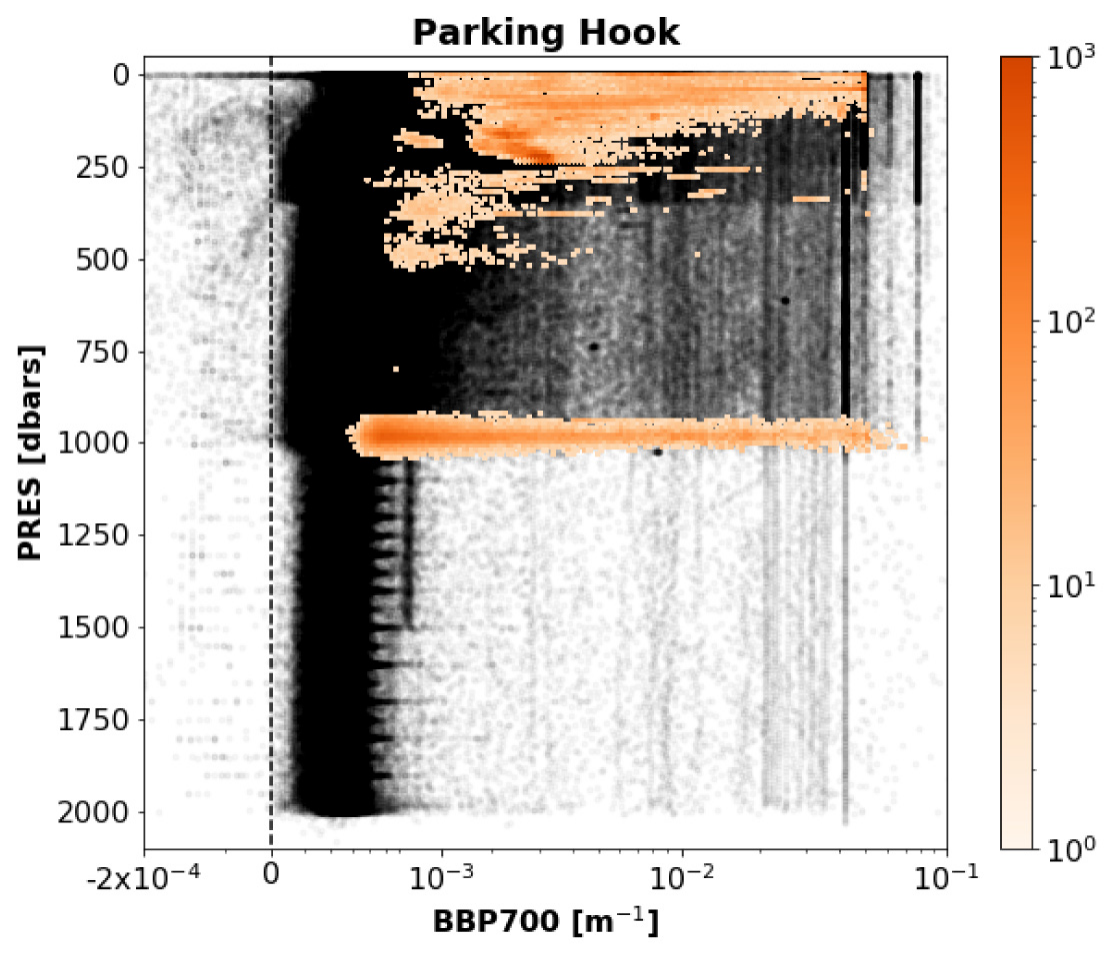
As Figure 3 but for the Parking-Hook test.

*Results*: This test flagged 0.4% of the current data in the GDAC. Although this is a relatively small number of points, these points represent a bias in the dataset that must be flagged. Figure 15 demonstrates that test flagged points near the standard parking pressure of 1000 dbar, but also several points from floats that were parked at considerably shallower depths.

### Test overlap

**Figure 16.**
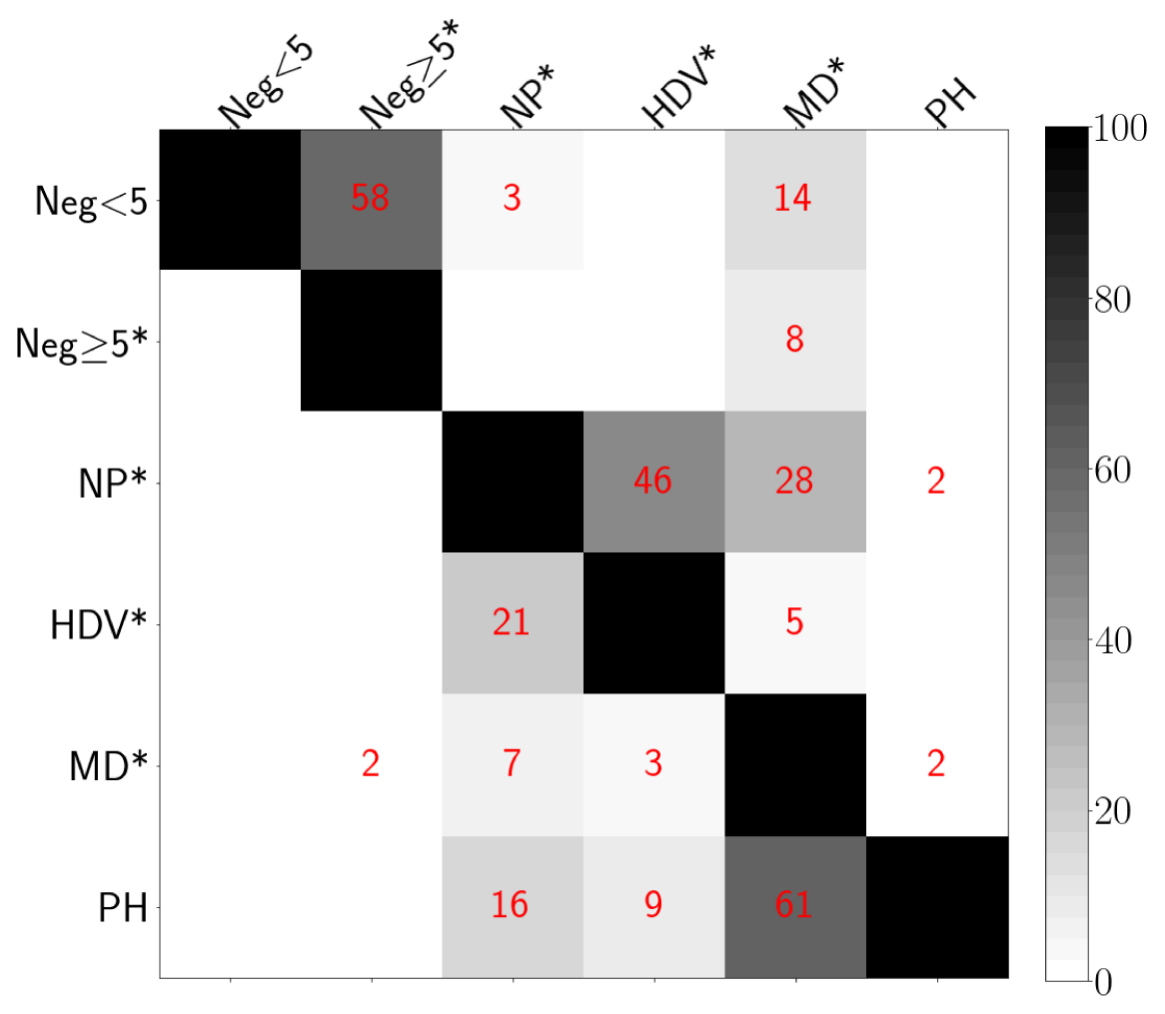
Percent overlap between pairs of different tests. Test labels as follows (* indicates a test that flags the entire profile): Neg<5: Negative BBP only within the upper 5 dbar; Neg≥5*: Negative BBP deeper than 5 dbar; NP*: Noisy Profile; HDV*: High Deep Value; MD*: Missing Data; PH: Parking Hook.

Figure 16 presents a matrix with the percentage of points from the entire GDAC dataset that were flagged by pairs of tests. Values were computed as the number of points flagged by each pair of tests, divided by the number of points flagged by the test with row label (lower left side of the matrix) or by the test with column label (upper right side of the matrix). To help the reader interpret the values presented in Figure 16, we provide the following example: 2% of the points flagged by the Missing-Data test were also flagged by the Parking-Hook test, while 61% of the points flagged by the Parking-Hook test were also flagged by the Missing-Data test.

### Impact of RTQC tests on GDAC BBP data

**Figure 17.**
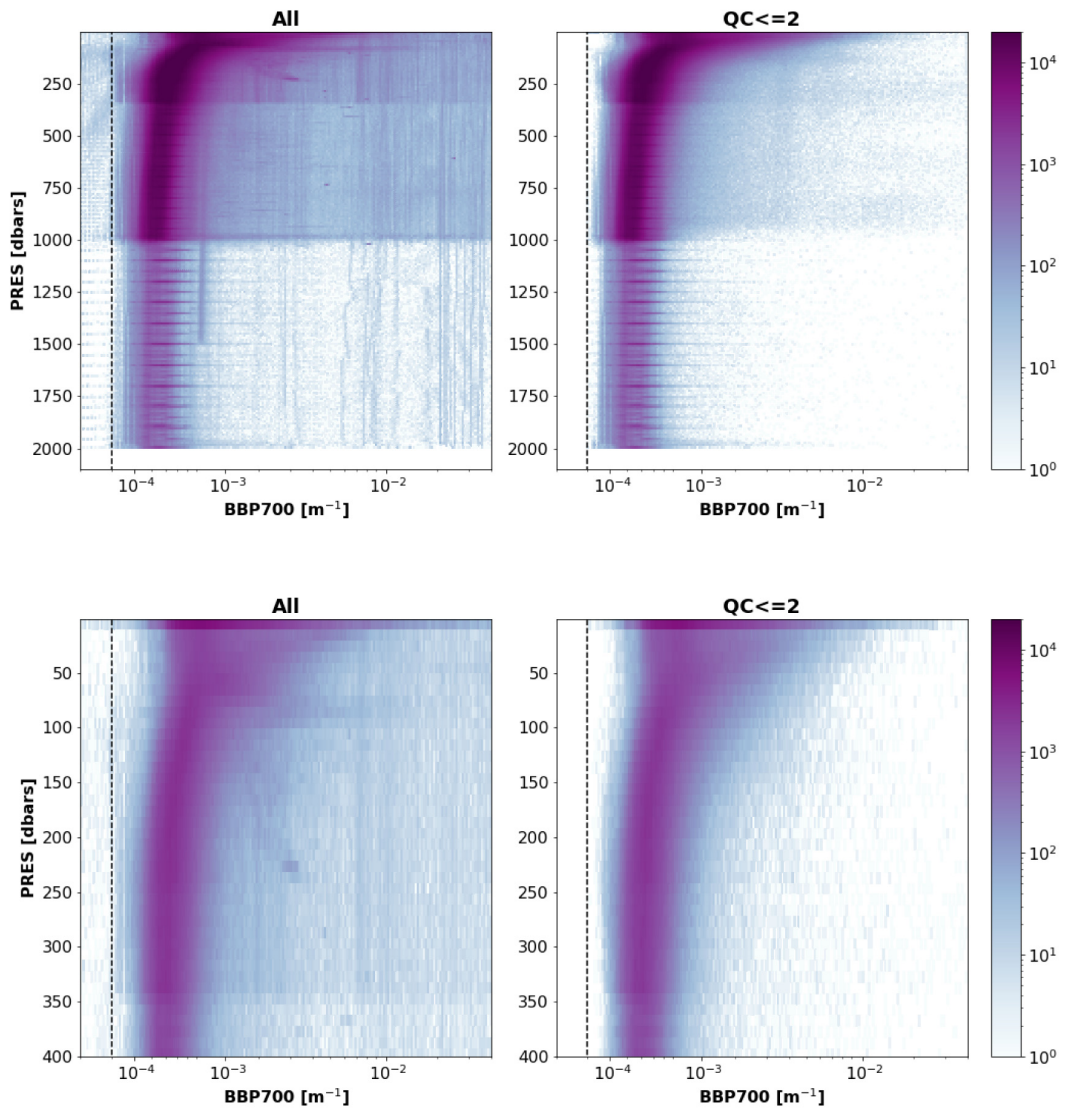
Two-dimensional histograms of the analysed raw and quality-controlled BBP data. Left plots: All current GDAC BBP data. Right plots: Data with QC<=2 resulting from implementing the new RT QC tests. Top and bottom rows present the same data but between 0 and 2000 dbar and 0 and 400 dbar, respectively.

The new RTQC tests proposed above assigned a QC flag >2 to ~19% of the BBP data points analysed and improved the shapes of the remaining profiles relative to expectations ( Figure 17). For example, negative values and profiles with consistently high values at depth were removed, and so were high BBP values near parking depths (e.g., 1000 dbar).

### Plans for recording the results of the tests

Understanding which BBP-RTQC tests have failed is needed to diagnose the quality of a BBP profile and to implement further DMQC tests. We have therefore started devising a method to record this information in the BGC-Argo files. However, to achieve this while maintaining consistency in file formats across DACs, we first need to find an informal agreement among the Argo DACs and then obtain official approval from the Argo Data Management Team. Therefore, it is impossible at the moment to provide further specifications about how exactly this will be achieved.

## Discussion

### Comments on overall results of these BBP RTQC tests

The proposed RTQC tests removed most of the anomalous BBP profiles ( Figure 17) and improved the overall quality of the BBP dataset, thus making it more suitable to be exploited by users. These tests assigned a QC flag >2 to ~19% of the BBP data points currently present in the GDAC. To ensure that the user can understand the history of the quality control applied to BBP data, pass/fail results of the proposed tests will be stored as a cumulative binary flag in the Argo NetCDF file (specifics will be provided in the Argo BBP quality control manual, when it will become available).

### Comments on selected proposed tests

***Missing-Data test*.** The Missing-Data test flagged the largest number of BBP data points because a relatively large fraction of shallow profiles are present in the global data set, due to the initial exploratory phase of the BGC-Argo programme. An additional reason for the large number of flagged data is that this test flags the entire profile, rather than specific points in a profile.

The rationale for defining this rather strict flagging procedure is that the main way in which we can identify faulty BBP values in real time is to inspect values of BBP at depth (with the High-Deep-Value test). Deep values are expected to be relatively small and stable with respect to surface values and can thus be used as a reference to quality control the rest of the profile. If these deep data are not collected, then these important reference values are not available to support the RTQC. Therefore, we decided to assign a QC flag of 3, so that shallow profiles can be re-assessed more carefully during the DMQC.

A more complex test was initially devised to overcome the above limitation, but feedback from the Argo community suggested that the Missing-Data test should be kept as simple as possible, in order to avoid overburdening DACs with implementing overly complex tests.

It is envisioned that, during Delayed-Mode Quality Control, shallow profiles could be easily re-qualified as “good data” if floats also collected at least some deep profiles. In other words, when a float has collected both shallow and deep profiles, the DMQC flags of the deep profiles could be extended (after inspection) to the shallow profiles as well. Alternatively, a delayed-mode operator may have other means to requalify data points that were flagged during the real-time quality control (e.g., comparison to climatologies).

***High-Deep-Value test*.** The High-Deep-Value test is based on the assumption that deep BBP values are low and stable, as it is often the case in the open ocean. As a consequence, this test flags profiles with high values at depth, even if these high values are real. Specific examples include floats that “grounded” (i.e., that touched the sea floor) and floats that sampled high BBP values at depth near continental margins or rivers. A first inspection of the flagged profiles, however, indicates that these specific examples are a relatively small fraction of the profiles flagged by this test.

BBP profiles of grounded floats could be identified in DMQC with the help of bathymetric maps, but again, such operation was deemed too complex for RTQC. Similarly, additional information on bathymetry and rivers could be employed to screen, during DMQC, floats that sampled close to the continental margins. It is thus a test where flags can be reversed in DMQC after careful evaluation of the circumstances (e.g., trajectory and sampling pattern) of the float.

In the future, BBP sensors may also be deployed on Deep-Argo floats (i.e., Argo floats specialised in sampling the entire water column, down to 6000 dbar) to measure sediment resuspension in the bottom boundary layer of the ocean. In this case, the High-Deep-Value test will have to be revisited to only use data in the upper water column (700-2000 dbar). This is not a problem for Argo, yet.

***Noisy-Profile test*.** The Noisy-Profile test was developed and tuned to flag profiles affected by noisy data. Because this test relies on detecting a certain percent of outliers, it could flag profiles containing real spikes ( Briggs *et al.*, 2011; Haëntjens *et al.*, 2020). We therefore recommend users interested in implementing spike analyses to use the raw BBP profiles.

### Overlapping tests

Some of the tests proposed flagged a significant number of common data (e.g., High-Deep-Value vs. Noisy-Profile and Parking-Hook vs. Missing-Data, Figure 16). Nevertheless, in keeping with our “conservative philosophy” of removing most of the bad data, we have decided to use all five tests proposed. This is because only when applied together were these tests able to generate a satisfactory RTQC BBP dataset.

### Potential additional BBP RTQC tests

After implementing the proposed BBP RTQC tests at the DAC level, we envision that additional RTQC tests could be proposed to further improve the quality of the dataset.

One potential future test that could be developed is a Regional-Range test. As the BGC-Argo BBP dataset grows in size, it should become possible to define and tune the parameters of a range test to specific ocean regions and specific seasons of the year. These tuned BBP-range parameters could be used in a Regional-Range test that can deliver better RTQC BBP profiles based on local conditions. It remains to be seen if such a test would be useful.

Another test that could potentially improve the overall quality of the dataset is the Animal-Spike test. Under certain conditions, mesopelagic organisms can be attracted to the light emitted by the optical sensors mounted on BGC-Argo floats, causing large localised spikes in BBP and other optical signals. Haëntjens *et al.* (2020) developed a detailed procedure to detect these events that could be implemented as a separate BBP RTQC test. As a first step and to avoid increasing the complexity of the proposed tests, we decided not to include this specific test, partly because the Noisy-Profile test already detected some (although not all) profiles affected by animal spikes. Nevertheless, future developments in BBP RTQC could add this test. Animal spikes are real signals that, however, may not be useful to many non-expert users (e.g., focusing on using BBP to estimate particulate carbon concentrations). We have therefore also identified the need to define a specific DMQC flag for this type of data.

Finally, as the proposed tests are implemented and users begin exploiting the RTQC BBP dataset, we expect that imperfections in the tests will be identified, which will result in further tuning of the test parameters.

### Adjusting BBP after RTQC

Argo variables that have been quality-controlled and that have received a correction are typically stored in corresponding “adjusted” variables (e.g., BBP_ADJUSTED). Argo has spent efforts to educate its users to select adjusted variables as the best available Argo data. Although the presented RTQC tests for BBP do not apply corrections to the BBP dataset, following discussions with the Argo community, we decided that DACs should create a BBP_ADJUSTED variable by applying to real-time quality-controlled BBP data a linear equation with OFFSET=0 and SLOPE=1. In other words, BBP and BBP_ADJUSTED variables will be equal. The rationale behind this choice is that non-expert users have been trained to use Argo adjusted variables as the best available Argo data. Our choice therefore aims at delivering a consistent message to the users. Until the delayed-mode quality control of the BBP data has been implemented, we also decided that no error field will be filled for the BBP_ADJUSTED variable.

## Conclusions

A new set of real-time quality-control tests for Argo BBP profiles was presented. When implemented, these tests will deliver a BBP dataset that is quality-controlled so that non-experts can use the BBP data in real time. Results of these tests were generated for the entire BBP dataset held at the GDAC and extensively discussed with the interested Argo community. The tests were approved by the BGC-Argo Data Management Team in December 2021. Furthermore, the same tests could also be adopted by or adapted for other measuring networks such as ship-borne or glider measurements.

As discussed, there may be cases where profiles subject to the RTQC tests outlined herein are erroneously flagged. Such profiles could be easily identified with the adopted flagging scheme and then reviewed and potentially recovered by a delayed-mode operator. Additional methods in support of delayed-mode quality control are also currently under development, including semi-annual audits on the global BBP array via comparative analysis against a machine-learning product ( Sauzéde *et al.*, 2020).

The final proposed tests resulted from a compromise between i) generating a quality-controlled BBP dataset in real time, ii) assigning flags that help the DM operators, and iii) avoiding burdening DACs with overly complicated tests. The Python code for the tests as well as example inputs and expected outputs for each test have been provided to facilitate implementation at the DAC level.

## Ethics and consent

Ethical approval and consent were not required.

## Data Availability

The original Argo data used in this study (snapshot of December 2021) are freely available in NetCDF format from: https://www.seanoe.org/data/00311/42182/#90179 This dataset is available under the terms of the Creative Commons Attribution 4.0 International license (CC-BY 4.0).

## Software availability

Source code is available from: https://github.com/euroargodev/BBP_RTQC/releases/tag/BBP_RTQC_v2.0.0

Archived source code at time of publication: https://doi.org/10.5281/zenodo.7934400

License: MIT
