# Supplementary material for: Real-time quality control of optical backscattering data from Biogeochemical-Argo floats
Source: Open Res Eur. 2023 May 30;2:118. Originally published 2022 Oct 13. [Version 2] doi: 10.12688/openreseurope.15047.2 (PMC10446022; doi:10.12688/openreseurope.15047.2)
Supplement: Peer review history

# Peer review: Reviewer response for version 2

**DOI:** 10.21956/openreseurope.17363.r34187

**Published:** 2023-09-05

**Authors:** Carolina Amadio

**Affiliations:** 1 Istituto Nazionale di Oceanografia e di Geofisica Sperimentale OGS, Sgonico, Italy

The paper introduces a new quality control procedure for optical backscattering data measured by BGC-Argo. Five tests are chosen to obtain the “best available Argo data” in real time, labeled as Adjusted Mode (AM). The document can be particularly useful for both the Argo community and non-expert users, as it is clear and well structured.

**General comments:**

- I appreciate the choice to present QC tests using a common structure (objective, example, implementation, flagging, flow_chart, results).
  However, I would expect to receive similar information for all tests in each of the sections. For example, in the “High-Deep-Value test” the choice of a threshold is explained in the "Objective" section, while in the other tests the thresholds are explained in the "Implementation" section. In the section ‘‘Implementation’’ of the ”Negative-BBP-test”, I would expect to find the information that the test is performed at 2 depths/layers (as for the other tests).
- Regarding the use of the thresholds, it is understandable that they are based on visual inspection and/or expert assessment. It would be helpful to better explain the choice of the thresholds used (e.g. like it is done for the “High-Deep-Value test”). Furthermore, did you evaluate the impact of using different thresholds? Have you considered to statistically define the thresholds?
- It might be useful to summarize the information and the results of the tests in a table. Here is an example:
  in rows the 5 tests, in columns: 
  Depth/layer (m) || Threshold (m-1) || Nr. of points || QC(s)* || % discarded
  *QS --> specifying whether a label refers to the entire profile or to single points

- If I understand correctly, the Missing Data-test assigns QC=3 to all profiles shallower than 895-1000 dbar (the last bin). Does this mean that no real-time data are available in areas of the ocean where bathymetry is < 895 dbar (e.g., regional seas)?
  If the answer is yes:
  (i) I suggest specifying this problem as a test limitation for shallow basin/areas
  (ii) in the High-Deep-Value test the condition “is the profile deeper than 700 dbar” becomes useless. 
  (iii) in the Noisy-BBP test the condition “Are there at least 10 points in this profile?” become useless
  If the answer is no:
  (i) Highlight what happens in cases of shallow bathymetry.
- Test overlap: The main purpose for which you decided to implement the test is not completely clear from the text. On the one hand, the overlap test can be useful to confirm the presence of anomalous profiles, demonstrating the robustness of your tests choice. On the other hand, however, it could be used as an indicator of test redundancy. If I understand correctly, which of the two aspects (confirm the test/avoid redundancy) do you consider more relevant?
- The RTQC uses 4 different values of QC (1, 3, 4, 9). It can probably be 
  useful to list them at the beginning of the “Results” Section, also explaining why QC=2 is never used (since QC=2 appears in the table 1 and figure 17 )

**Detailed comments (minor):**

 Introduction:

- Add bbp unit of measurements at the beginning of the introduction .
- “Argo’s objective is to sustain 1000 operational six-variable BGC-Argo floats in the global ocean” - list variables or remove sentence. “So far more than 600 BGC-Argo floats have been equipped with optical backscattering sensors, and ~250 of them are currently active.” I suggest reversing the order of the two sentences.
- “now obsolete, given the new tests presented in this work” Why are the tests obsolete? I suggest to explain better or remove the sentence.

Results:

- “Objective, presenting the purpose of the test” I suggest adding “presenting the purpose of the test and the BGC-Argo target problem(s) to be addressed” (e.g. malfunctioning of sensor etc.).
- High-Deep-Value test: I suggest moving the explanation of the threshold value to the “Implementation” section.
  Furthermore, I find a slight discrepancy between the order of magnitude of the threshold value (10-4 m-1) and the x-axis label (10-2) in Figure 4. It can be solved by making explicit in the text "the blue line in Figure 4" when introducing the threshold value or by changing the x-axis label in Figure 4.
- Parking-Hook test: I suggest rephrasing the "Implementation" part, reducing the information. For example: “The test is applied to all ascending profiles when the distance (LAYER1) between the last 2 points of a profiles is less than 20 meters (G_DELTAPRES2) and the distance (LAYER2) between the parking pressure and the maximum depth (maxPRES) is less than 100dbar. The median between BBP at LAYER1 and LAYER2 is added to a threshold (G_DEV = 0.0002 m−1). The test fails whenever data in LAYER2 are higher than the computed baseline.

Discussion:

- “Finally, as the proposed tests are implemented and users begin exploiting the RTQC BBP dataset, we expect that imperfections in the tests will be identified, which will result in further tuning of the test parameters.” By using "further" in the previous sentence, I understand that you have tuned the parameters, but I do not see any reference to this in the “Results Section".

Is the rationale for developing the new method (or application) clearly explained?

Yes

Is the description of the method technically sound?

Yes

Are the conclusions about the method and its performance adequately supported by the findings presented in the article?

Yes

If any results are presented, are all the source data underlying the results available to ensure full reproducibility?

Yes

Are sufficient details provided to allow replication of the method development and its use by others?

Yes

Reviewer Expertise:

BGC-Argo dataset and BGC-Argo data assimilation in operational models

I confirm that I have read this submission and believe that I have an appropriate level of expertise to confirm that it is of an acceptable scientific standard.

# Peer review: Reviewer response for version 2

**DOI:** 10.21956/openreseurope.17363.r33065

**Published:** 2023-08-10

**Authors:** Sandy J. Thomalla

**Affiliations:** 1 Southern Ocean Carbon and Climate Observatory (SOCCO), CSIR, Rosebank, South Africa

**Review: Real-time quality control of optical backscattering data from Biogeochemical-Argo floats**

 Sandy Thomalla

 This manuscript presents a suite of 5 real-time quality control tests for application to optical backscattering profiles in the BGC-Argo data set. The paper is well written and clearly explains the structure of the tests, why they are needed, how they are implemented and the impact that their implementation has on the data set. I particularly liked the flow charts which I found especially clear (and indeed in some cases easier to follow than the “Implementation” text). These methods are needed by the growing user community that is interested in carbon flows, in particular those wanting access to real time data that is appropriately quality controlled to exclude bad or suspect data. 

 I find this manuscript suitable for publication with only minor comments listed below. Without line numbers it was challenging to identify their specific reference, so in addition I have included all comments on the attached .pdf with the location of their relevance highlighted in green. I have chosen to sign my review as I am an advocate for transparency and accountability of review.

**Introduction.**

 It is maybe worth highlighting that the typical bbp wavelength of the BGC argo data base is 700nm? And why? And what alternate wavelengths are typically implemented?

 I think that it is worth adding that in particular bbp can be used as an alternate proxy for phytoplankton biomass that is not impacted by quenching. I would suggest highlight that optical observations such as backscatter are better correlated to carbon than chlorophyll and that they can provide independent measures of phytoplankton biomass in open ocean waters (away from regions with highly scattering inorganic material). Unlike chlorophyll, bbp is more likely to be insensitive to changes in the intracellular concentration of pigments. In addition, the ratios between chlorophyll and bbp can be used to correct for quenching and in addition, chl:bbp ratios can be used to infer and elucidate on possible physiological adjustments to environmental stressors and community structure (i.e. i would recommend providing additional motivation for why the variable bbp is useful to the community). 

 Add parenthesis (RT) and (DM) to the first use of Real Time and Delayed Mode. 

 I am curious as to why you highlight non-experts per se (here and elsewhere). I appreciate that so called experts would be more inclined to examine the data and post-process it allowing for corrections to be applied where appropriate (e.g. a revised dark count to prevent negative offset etc.). But to me the key user here is anyone who needs to access near real-time data (regardless of their level of expertise). You provide one example of a non-expert user as an operational modeller, could you possibly elaborate on this further? Specifically, are you able to highlight the benefits to operational modellers of having access to real time data instead of delayed mode data (especially considering that delayed mode data are generally considered “better” having undergone the QC scrutiny in addition to the application of corrections where possible).

 Are the two tests listed for bbp in the Argo Quality control manual currently being implemented to the ARGO data set? i.e. is some level of QC being implemented and data being flagged, albeit that these 5 tests are considerably better?

 Can you possibly elaborate on the obsolete global rage and spike tests please? I appreciate that the ensemble of tests being presented here is better, but I think that it is worth elaborating on why the current tests are inadequate (how do they work?/ why don’t they work well?). If those obsolete tests are being ineffectually implemented you could also compare the % of flagged data from the implementation of the current 2 tests versus your suite of 5 (to highlight how much suspect data was passing QC). A slight elaboration on this would provide additional motivation and rationale for the relevance of the tests being proposed in this manuscript and the requirement for them to be implemented as standard procedure. 

**Data and Methods**

 ​​Hyphenate non-experts (as per other references)

 The reason for number 4) of minimising impact to the DAC’s is not clear to me (i.e. providing input and expected output for each test). Is this for comparison to your application to ensure that it is being applied correctly? Maybe this point could be made clearer, or elaborated or even possibly excluded? 

**Approach**

 Can you please clarify that to define the new BBP RTQC tests, you followed an iterative process of community engagement. When I first read this sentence I thought it was referring to the iterative application process of one QC step after the other (which is not I think what you meant in this instance). 

 The ‘in vacuo’ in this instance is confusing me. Since the 5 RTQC tests were actually developed for the bbp measurements made in situ by BGC-Argo at 700nm (i.e. not in a vacuum)? Albeit that θ, is measured at a given wavelength in vacuo. 

 Can you elaborate on how many other BGC-Argo floats there are with bbp at a wavelength other than 700? if so what is it typically? Also, why not test and show/confirm that the RTQC does apply to different wavelengths and works just as well at 700nm (as with any other wavelength)?

 Consider altering this sentence structure to read as follows (less negative). 

 “Interactions with the community were crucial in defining the final test suite. Although this approach introduced a certain level of subjectivity in how the tests were selected, it incorporated the knowledge of experts in optical backscattering and management of the Argo data stream providing fundamental input towards critically determining the final ensemble of test definitions.”

 Please clarify the statement regarding “even if profiles had been deemed bad by the DAC operators”? How were bbp profiles deemed bad by the DAC given that no real time QC on bbp was being implemented? Or were they deemed bad by the two obsolete QC steps mentioned earlier? 

 Your first step to minimise overlap is not clear to me. To me, determining the fraction of data points flagged by all pairs of tests does not minimise overlap. Especially if “All tests were applied independently of each other (no order was defined) and the statistics computed reflect this choice (i.e., the same data can be flagged by multiple tests).” Surely overlap would only be minimised if you instead applied the tests in series (and not in parallel) i.e. if a profile was deemed bad by the first test in the series it did not undergo the other 4 tests. 

**Results**

 “This order could be used to define the sequence in which the tests are applied during RTQC”. Indeed, were that approach taken it would understandably minimise implementation overlap by the DAC. 

 The following comment is true for all Implementation text regarding the use of words, code and units. I think it is appropriate that the code be included in parenthesis (but that it should exclude the units) but not that the thresholds are excluded from the text, which I think should be clear in the text and include the units. I would suggest that all “Implementation” sections be edited as per this example for the HIgh-Deep-Value test: 

 Implementation: This test first determines s fails if there are is at least 5 points a certain number (C_N_DEEP_POINTS = 5) of points deeper than a threshold depth of 700 dbar (C_DEPTH_THRESH = 700 dbar) and it fails if the median of the median-filtered profile below 700 dbar C_DEPTH_THRESH is greater than a predefined threshold of 0.0005 m-1 

 (i.e., C_DEEP_BBP700_THRESH = 0.0005 m−1).

 Consider including a possible reason for deep bbp values being an incorrect dark count (i.e. if underestimated). Which I believe can be quite common and easily rectified in DMQC.

 Add a space: to “good”

 Put (B_PRES_THRESH = 100) in brackets like the other code examples. 

 Please adjust as follows: The test fails if residuals with absolute values above a threshold value of 0.0005 m−1 (i.e., B_RES_THRESHOLD = 0.0005) occur in at least 10% of the profile data below the 100 dbar threshold. 

 Negative-BBP test could similarly occur if darkcount is too high, which is easily accounted for in DM processing to recover profiles.

 Pressure limit is missing from the code in brackets for second depth range test: “The test is then implemented over a second pressure range (i.e., PRES >= maxPRES - G_DELTAPRES1 = 20).”

 Figure 13: I was wondering if there is a way to identify the points that fall into both the 20mn and 50m depth ranges? Shouldn’t all dots in the bottom 50m of the profile be blue? (only three looking blue to me?). The two depth ranges that are compared to each other (via the mean) is not clear (to me) in the figure. 

**Discussion**

 By users … add “that access to require real time data”

 The second two sentences of the Discussion are repetitive (and just recently covered in the previous sections). 

 Adjusting BBP to RTQC. For what it is worth (and this is just my personal opinion here) I would have to disagree with the Argo community on this. If a non-expert user is after real time bbp data and there is none in bbp_ adjusted then they will by default fall back on using the unadjusted BBP data, which will have undergone RTQC and will have flags associated with it. As such, they will know that it has passed quality control measures but that it has NOT been adjusted in any way. I strongly feel that data should only be labelled as adjusted if it has indeed been adjusted e.g. by subtracting a revised dark count based on a percentile of deep data to compensate for incorrect dark values (as is applied for the chlorophyll in delayed mode processing).

Is the rationale for developing the new method (or application) clearly explained?

Partly

Is the description of the method technically sound?

Yes

Are the conclusions about the method and its performance adequately supported by the findings presented in the article?

Yes

If any results are presented, are all the source data underlying the results available to ensure full reproducibility?

Yes

Are sufficient details provided to allow replication of the method development and its use by others?

Yes

Reviewer Expertise:

Biogeochemistry, phytoplankton photophysiology, biological carbon pump, Southern Ocean, primary production, carbon export.

I confirm that I have read this submission and believe that I have an appropriate level of expertise to confirm that it is of an acceptable scientific standard.

# Peer review: Reviewer response for version 2

**DOI:** 10.21956/openreseurope.17363.r32321

**Published:** 2023-06-20

**Authors:** Griet Neukermans

**Affiliations:** 1 Biology Department, MarSens Research Group, Ghent University, Ghent, Belgium

I have no further comments.

 Griet

Is the rationale for developing the new method (or application) clearly explained?

Yes

Is the description of the method technically sound?

Yes

Are the conclusions about the method and its performance adequately supported by the findings presented in the article?

Yes

If any results are presented, are all the source data underlying the results available to ensure full reproducibility?

Yes

Are sufficient details provided to allow replication of the method development and its use by others?

Yes

Reviewer Expertise:

Particle backscattering, BGC-Argo data, biogeochemistry

I confirm that I have read this submission and believe that I have an appropriate level of expertise to confirm that it is of an acceptable scientific standard.

# Peer review: Reviewer response for version 1

**DOI:** 10.21956/openreseurope.16269.r30309

**Published:** 2023-01-30

**Authors:** Griet Neukermans

**Affiliations:** 1 Biology Department, MarSens Research Group, Ghent University, Ghent, Belgium

This paper clearly describes five real-time quality control tests applied to optical backscattering data from BGC-Argo floats. This paper is timely, given the rapidly growing non-expert user base of BGC-Argo bbp data and will be very useful to the BGC-Argo community and its users. The paper is well written and well structured, the data and results are of good quality, and the discussion is thorough. I recommend indexing of this work, but I have a few minor comments that may help improve the paper. I have provided my comments in comment boxes on the pdf paper which can be found here. I have listed them below for completeness and transparency:

 Minor comments:

- replace reflected by scattered. Reflection is only a part of light scattering. Please carefully check the definitions of backscattering and the VSF.

- remove "backward"

- wavelength in vacuo or in water?

- VSF by particles at a given angle

- and PIC see Balch *et al. *(1996) and Terrats et al. (2020) papers. ^1^^,^^2^

- Do QC flags values between 5 and 8 have any meaning? If yes, please specify

- replace with e.g.? the BGC-Argo community interested in the quality control of BBP is probably wider than the list of co-authors of this paper.

- Wavelength in vacuo

- Correct: this repository if the the first author

- new, with respect to what?

- I suggest to add a figure with a decision tree for each of the QC tests for quick and easy visualization of the tests and QC flags.

- what is the rationale for defining these bins?

- please give range for "considerably lower"

- because of the high overlap between flagged data and non-flagged data it may be better to use density plots for the entire dataset, showing the number density of points with and without flags. Same comment to Fig 2 , 4, 6, 8 , and 10.

- Perhaps useful to say how these spikes differ from spikes associated with large particles that are not large organisms

- do you mean absolute values? as the residuals can be negative because of the median-filtering

- is there an explanation for these two vertical lines?

- useful to specify which expectations you mean

- but see seasonality in Poteau et al. 2017. ^3^

- meaning of "grounded"? Touched the seafloor?

- also add Haentjens ref

- Again, all data can be plotted using density plots showing the number density of observations but data in the BBP adjusted variable are only BBP data with QC flag <2? Perhaps worth being explicit about what BBP_ADJUSTED i

Is the rationale for developing the new method (or application) clearly explained?

Yes

Is the description of the method technically sound?

Yes

Are the conclusions about the method and its performance adequately supported by the findings presented in the article?

Yes

If any results are presented, are all the source data underlying the results available to ensure full reproducibility?

Yes

Are sufficient details provided to allow replication of the method development and its use by others?

Yes

Reviewer Expertise:

particle backscattering, BGC-Argo data, biogeochemistry

I confirm that I have read this submission and believe that I have an appropriate level of expertise to confirm that it is of an acceptable scientific standard, however I have significant reservations, as outlined above.

## References

[1] : The 1991 coccolithophore bloom in the central North Atlantic. 1. Optical properties and factors affecting their distribution. *Limnology and Oceanography* .1996;41(8) : 10.4319/lo.1996.41.8.1669 1669-1683 10.4319/lo.1996.41.8.1669

[2] : Detection of Coccolithophore Blooms With BioGeoChemical-Argo Floats. *Geophys Res Lett* .2020;47(23) : 10.1029/2020GL090559 e2020GL090559 10.1029/2020GL090559 33380764PMC7757229

[3] : Particulate concentration and seasonal dynamics in the mesopelagic ocean based on the backscattering coefficient measured with Biogeochemical-Argo floats. *Geophysical Research Letters* .2017;44(13) : 10.1002/2017GL073949 6933-6939 10.1002/2017GL073949

# Peer review: Reviewer response for version 1

**DOI:** 10.21956/openreseurope.16269.r30306

**Published:** 2023-01-27

**Authors:** Gianluca Volpe

**Affiliations:** 1 Consiglio Nazionale delle Ricerche, Istituto di Scienze Marine (CNR-ISMAR), Rome, Italy

This is the revision of the paper “Real-time quality control of optical backscattering data from Biogeochemical-Argo floats” by Giorgio Dall’Olmo and co-authors. The manuscript describes five tests for the real-time quality assessment of automatically-acquired backscattering profiles from BGC-Argo platforms. The work is relevant not only for the Argo community but also for BGC-Argo users for which provides a valid background, as such it surely deserves publication in Open Research Europe. There are however a few remarks that I encourage the authors to take into consideration.

 A general comment is that the whole work looks too descriptive reading more like a meeting report (often highlighting the points of agreement) than a scientific paper. The five tests rely on thresholds whose definition is apparently based on subjective judgement, which for as intuitive and reasonable they can be they are still not supported by any scientific evidence or statistical analysis. This points to the question not explicitly addressed in the manuscript on whether DMQC is only applied to profiles initially flagged as 3 or to all profiles, independently of the RTQC. If all profiles do undergo DMQC independently of RTQC then the importance of RTQC is only relevant to applications needing real-time data (e.g., operational modelers). On the contrary, if DMQC is only applied to suspicious profiles determined by the RTQC, then it is important to determine the various thresholds in a more rigorous manner. To give the readers the flavor from one side of the importance of the expert review and on the other of the robustness of the general approach described in the manuscript, I would suggest the authors to color (or simply to provide percent numbers) the profiles shown in the various examples that effectively changed their status: for example, how many profiles were originally flagged as 2 or 1 and then turned 3 or 4 after expert judgement? And similarly how many profiles that were originally flagged as 3 or 4 actually turned 2 or 1 after expert judgement?

 Moreover, I understand that a quasi-binary (e.g., good vs no good data) flagging system is much easier to handle than a more complex system like the one adopted by the satellite data processing in which the flags surely provide a better means to quality assess the data. In this context, I do not see any reason for keeping things easier but rather to help users be more confident in data usage. One drawback of the proposed flagging system is that it does not allow users to discriminate data according to the various tests. This could also give useful feedbacks to the test developers.

 Going through the manuscript, I found curious and a bit frustrating as well that at the end of “Data and methods” it was still unclear what the tests are about and what are they aimed at. I would suggest the authors to reshape a bit the way the information is conveyed and in case to compact the relevant information about the five RTQC tests (e.g., thresholds, filtering application etc …) into a table that could be referred to.

 Information on the general Argo data handling approach could provide a context for non-expert users or for the non-community members and help them understanding what is behind the choice of simplicity or . For example, how frequent are the RTQC and DMQC testing? How many profiles the single DAC has to handle in terms of both testing (RTQC and DMQC)? How many DACs are involved?

 Here below more detailed comments on the various sections.

 The **abstract** is schematic and effective.

**Introduction**

*Since the Argo variable used to represent b _bp_ is BBP, we will use the latter in this manuscript.* – Non-expert readers may surely benefit from the addition of one sentence that explains the difference between the two bbps if indeed it exists. The way it is presented this sentence may create confusion, please rephrase it.

**Data and methods**

**Approach** section is not entirely clear and I personally find it a bit confusing. It refers to a series of details that surely provide the context in which the manuscript has developed but probably do not add any significant science to the paper. A better place where mentioning this kind of details would probably be the introduction. I would expect Data and methods to cover aspects that help the reader discriminating whether the tests are useful, scientifically sound and operationally feasible.

 Other things that I found confusing/not clear in this section are:

1. the tests that are often mentioned are not yet defined nor there is a link to any table/figure or section that the reader can promptly refer to: this is also mentioned in the general comments.
2. the authors refer to themselves as the community and this is done in a way as if the consensus reached among the coauthors of the manuscript should per se be a proof of the validity of the approach.
3. *These interactions with the community allowed us … - *this sentence does not add any particular or relevant information: that the coauthors/community of a work do interact among them is pretty obvious as it is obvious that they eventually reach an agreement.
4. It is not clear why the overlap among tests should be minimized. Having more than one test telling that the profile is not the best you might have measured is probably better, especially if the goal is to worn non-expert users on their usage. I suggest here to add a sentence to better explain why it is advisable that the tests do not overlap, if that is the case.
5. The link between the different sampling rate and the vertical resolution of the various sensors and missions with the need of smoothing the data with a median filter is not entirely straightforward. I can understand that for the sake of QC tests the application of a median filter to smooth the profile could be useful, advisable and foreseeable, but this should be properly justified.

Probably a better title for this section could be “background”.

**Results**

 Very often, to explain the various tests, English is substituted by a sort of programming language notation: although most of the times the meaning is intuitive it still distracts and one often has to go back and forth reading the same sentence to make sure the meaning is appropriately taken. I recommend the authors to use English and where necessary or helpful to add the “programming language” notation. I found this particularly true in correspondence of the implementation of the parking-hook test.

**Missing-Data test**

 Since the 10 bins are quite large (50 to 100 m), I would expect data abundance per bin to be higher, so perhaps MIN_N_PERBIN should be set larger than 1 according with the rate of acquisition and the float vertical velocity.

**High-deep-value test**

 My understanding is that the rationale for the high-deep-value test is to spot profiles affected by any kind of sensor issues. In this view, it would probably make sense, once a profile is flagged, to also look at the temporal variability of the closest profiles acquired with the same float. Similarly, the overall shape of the profile should somehow suggest whether the profile should be flagged as 2 or 4, thus removing the need of the expensive expert judgement.

 Right out of my curiosity (other readers could find it interesting as well), how would the profile of Figure 3 be flagged by an expert? At a very first sight the profile looks absolutely reasonable but probably affected by a bias depending whether or not it was acquired in a high productive area.

**Noisy-Profile test**

 Why is this test based on the absolute residuals and not over a percent or relative units threshold? The percent threshold is probably easier to implement especially if the test is meant to be applied to all sensors deployed globally.

**Parking-Hook test**

 The implementation part should be rewritten. Many times the authors refer to variables that have not previously defined making the reading heavier than necessary. Similarly, as already mentioned, the authors should write in proper English avoiding coding language where possible. The addition of equations could go in the right direction.

**Test overlap**

 Before reading about the example provided by the authors to interpret figure 11, I understood that the test overlap was computed over single measurements (points). Then I wonder, how can a data point fail both the missing data test and the parking hook test, especially because the missing data test is applied over a depth range totally different than the parking-hook test? I am confused perhaps because I still don’t understand the point of considering the test overlap. One consideration is that perhaps there should be two different flagging systems: one for the profile and the other for the single measurement. Moreover, the authors may want to consider the additive flagging system method used, for example, in the Level-1 to Level-2 satellite data processing. The advantage of this method is that each test has its own value which can then be added to the others and independently of the others; the result is that pixel (data point in this case) can be flagged with and thus sorted according to any of the applied tests.

**Discussion**

**Comments on selected proposed tests**

 One important remark is about the authors’ choice (driven by the Argo community feedback) of keeping the various tests as simple as possible even if more complex and likely more robust tests can be envisaged also in real-time. These tests should be as robust and reliable as possible with the general aim of minimizing as much as possible the expensive human intervention. Given the general simplicity of the shown tests, it is hard to see how a “more complex” test could overburden DACs. The point here is to operationally run the RTQC procedure (i.e., a python script?) to assign a specific value to the profile or to each of its data points. This has little or nothing to do with the complexity of the test which could also take account of the local bathymetry or climatology, which could and actually should be generated at GDAC level and disseminated to local DACs. Lack of ancillary data at the time of RTQC appears a much solid reason for not running the test, not simplicity.

**Missing-Data test.**

*An additional reason for* … - this is connected to one of my previous comments on the need of either splitting the QC flagging system into two (profile and single data record) or to adopt an approach similar to satellite data processing.

**High-Deep-Value test**

 I do not see any inconvenience nor complexity in using the bathymetry also in real-time quality testing.

Is the rationale for developing the new method (or application) clearly explained?

Yes

Is the description of the method technically sound?

Yes

Are the conclusions about the method and its performance adequately supported by the findings presented in the article?

Yes

If any results are presented, are all the source data underlying the results available to ensure full reproducibility?

Yes

Are sufficient details provided to allow replication of the method development and its use by others?

Yes

Reviewer Expertise:

optical oceanography

I confirm that I have read this submission and believe that I have an appropriate level of expertise to confirm that it is of an acceptable scientific standard, however I have significant reservations, as outlined above.
